# Protective effects of salvianolic acid A on ischemic stroke: A meta-analysis of preclinical studies

**DOI:** 10.3389/fphar.2025.1629258

**Published:** 2025-11-19

**Authors:** An Liu, Wenjing Li, Hangyao Zhang, Kexin Sun, Yuqian Xiao, Jun Wan, Pin Li, Yan Wang, Sihan Hu, Yanjie Li, Yanjie Bai

**Affiliations:** 1 Rehabilitation Centre of the First Affiliated Hospital of Henan University of Chinese Medicine, Zhengzhou, China; 2 Rehabilitation Medicine College, Henan University of Chinese Medicine, Zhengzhou, China; 3 The First College of Clinical Medicine, Henan University of Chinese Medicine, Zhengzhou, China; 4 Department of Integrated Traditional Chinese and Western Medicine, Peking University First Hospital, Institute of Integrated Traditional Chinese and Western Medicine, Peking University, Beijing, China; 5 Department of Rehabilitation, Henan Provincial Hospital of Traditional Chinese Medicine, Zhengzhou, China

**Keywords:** salvianolic acid A, ischemic stroke, ischemia injury, preclinical evidence, meta

## Abstract

**Objective:**

The aim of this study was to investigate the protective effects of salvianolic acid A (SalA) on cerebral ischemic injury following ischemic stroke (IS) and its possible mechanisms, providing a scientific basis for future clinical research on IS.

**Methods:**

A comprehensive search was conducted across eight databases: PubMed, Embase, Web of Science, the Cochrane Library, China National Knowledge Infrastructure (CNKI), Wanfang Database, VIP Database, and the China Biomedical Literature Database (CBM). The search included all literature from the establishment of each library up to February 2025. Data analysis was performed using STATA 15.0 software.

**Results:**

A total of 15 studies involving 564 animals were included. The analysis showed that compared to the control group, SalA significantly reduced the infarct volume [*SMD* = −4.67, *95% CI* = (−5.98, −3.36), and *p* < 0.001] and brain edema area [*SMD* = −5.291, *95% CI* = (−7.607, −2.975), and *p* < 0.001] and improved neurological deficits [*SMD* = −6.39, *95% CI* = (−9.091, −3.688), and *p* < 0.001]. SalA also significantly inhibited interleukin 6 (IL-6), tumor necrosis factor-a (TNF-α), IL-1β, and other indicators, such as Bcl-2-associated X protein (Bax) and Caspase-3 index, while showing a positive effect on B-cell lymphoma-2 (Bcl-2), Bcl-2/Bax, and other indicators.

**Conclusion:**

This meta-analysis demonstrates the therapeutic effects of SalA on IS. The results indicated that SalA significantly reduced the infarct area, improved neurological function scores, and alleviated brain edema. These effects were achieved through multiple mechanisms, including anti-inflammatory, antioxidative, antiapoptotic actions, along with blood–brain barrier (BBB) repair. SalA exhibited dose-dependent effects at different doses (especially 20 mg/kg) and administration methods. Further high-quality preclinical and clinical studies are needed for analysis.

**Systematic Review Registration:**

identifier INPLASY2025110038

## 1 Introduction

According to the Global Burden of Disease (GBD) 2023 study, stroke is the second leading cause of death and the third leading cause of death and disability combined worldwide. From 2010 to 2023, the absolute burden of stroke increased substantially, with incident cases increasing by 70.0%, prevalent cases by 86.0%, deaths by 44.0%, and disability-adjusted life years (DALYs) by 32.0%. Ischemic stroke (IS) is the most common subtype, accounting for approximately 65.3% of all new strokes in 2023 (Hay et al., 2025). The pathophysiology of IS is initiated by the interruption of cerebral blood flow, typically due to thrombosis or embolism, leading to a cascade of detrimental events, including energy failure, excitotoxicity, oxidative stress, inflammation, and apoptosis, which ultimately cause irreversible neuronal damage (Cheng et al., 2024).

Current standard treatments for acute IS, such as intravenous thrombolysis with recombinant tissue plasminogen activator (rt-PA) and endovascular thrombectomy, are primarily focused on recanalization. However, these therapies are constrained by a narrow therapeutic window and the risk of hemorrhagic complications, leaving a significant portion of patients ineligible or with poor outcomes. Moreover, even with successful reperfusion, the subsequent ischemia–reperfusion injury (IRI) often triggers a secondary wave of damage, including neuroinflammatory storms and blood–brain barrier (BBB) disruption, for which effective clinical interventions remain limited. This therapeutic gap underscores the urgent need for novel neuroprotective agents that can target multiple pathological pathways and extend the window for treatment (Liang et al., 2023).

**FIGURE 1:**
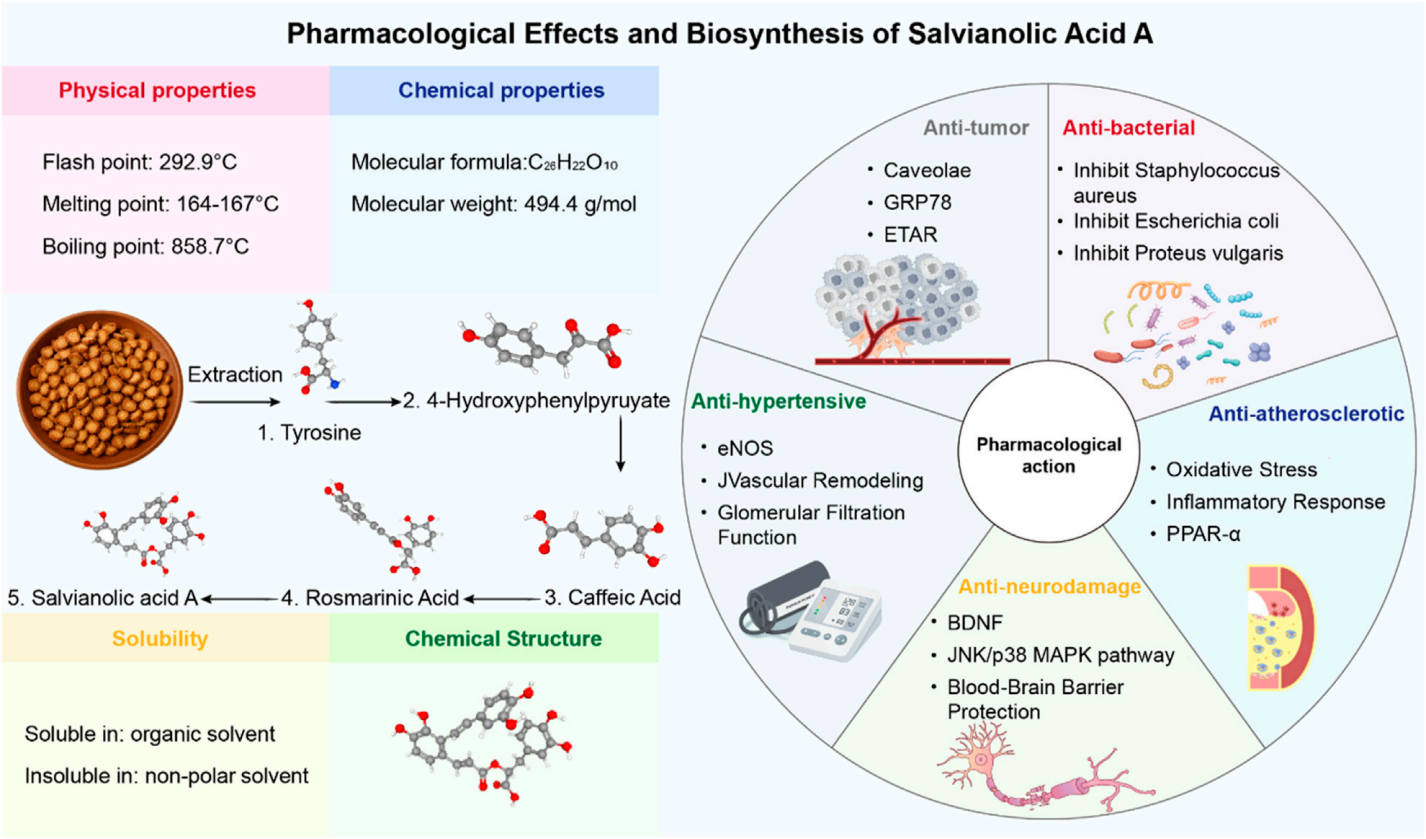
Pharmacological effects of salvianolic acid A.

In the search for such multi-target therapeutic strategies, natural products derived from traditional medicine have emerged as a promising resource for drug discovery (Hao et al., 2024; Hu et al., 2022; Niu et al., 2024). Salvianolic acid A (SalA) is a prominent water-soluble phenolic acid extracted from *Salvia miltiorrhiza* Bunge (Danshen), a perennial herb widely used in East Asia for treating cardiovascular and cerebrovascular diseases (Xd et al., 2019). As the core active ingredient in the approved Danshen polyphenolic acid salt injection, SalA has garnered significant attention for its potent biological activities, including anti-inflammatory, antioxidative, and antiapoptotic effects (Li et al., 2020). Preclinical studies have shown that SalA can cross the BBB and exert neuroprotective effects in various models of cerebral ischemia (Pang et al., 2016; Figure 1).

Despite these promising findings, the existing preclinical evidence for SalA’s efficacy in IS is scattered across numerous studies with considerable methodological heterogeneity. These studies vary widely in terms of the animal species and models used [e.g., middle cerebral artery occlusion (MCAO), tMCAO, and photothrombotic models], the dosages and administration routes of SalA, and the outcome measures evaluated. Such inconsistencies make it challenging to draw a firm conclusion about the overall therapeutic potential of SalA and to guide the design of future clinical trials. Therefore, this study was conducted to perform the first systematic review and meta-analysis of preclinical studies to quantitatively synthesize the available evidence on the protective effects of SalA in animal models of ischemic stroke, explore the sources of heterogeneity, and provide a more robust assessment of its potential as a therapeutic agent for IS.

## 2 Materials and methods

The meta-analysis was conducted according to the Preferred Reporting Items for Systematic Reviews and Meta-Analyses (PRISMA) guidelines (Page et al., 2021).

### 2.1 Retrieval policy

A comprehensive and systematic literature search was conducted across eight electronic databases: PubMed, Embase, Web of Science, the Cochrane Library, China National Knowledge Infrastructure (CNKI), Wanfang Database, VIP Database, and the China Biomedical Literature Database (CBM). The search covered all articles published from the inception of each database up to 28 February 2025.

The search strategy was developed to be highly sensitive, combining controlled vocabulary terms (e.g., MeSH and Emtree) with free-text keywords related to two core concepts: “salvianolic acid A” and “ischemic stroke.” The search strategy for PubMed is provided as an example: (((“Salvianolic Acid A” [MeSH Terms] OR “SalA” [All Fields])) AND ((“Ischemic Stroke” [MeSH Terms] OR “Brain Ischemia” [MeSH Terms] OR “MCAO” [All Fields]))). This strategy was adapted for the syntax of each of the other databases. For Chinese databases, the search strategy utilized the Chinese translations of the keywords. The complete, detailed, and reproducible search strategies for all eight databases are provided in Supplementary Material l.

No language restrictions were applied during the initial search phase, but only articles published in English or Chinese were included for full-text screening. Two authors independently performed the literature search, and the results were cross-checked to ensure completeness.

### 2.2 Eligibility criteria

The inclusion criteria are as follows: (1) studies involving rats or mice as subjects, with the establishment of MCAO or I/R models; (2) the experimental group receives SalA intervention post-surgery (selecting the group with the best therapeutic effect if different concentrations are used); (3) the model group receives a placebo or no treatment; (4) no restrictions on animal species, gender, age, weight, or sample size; (5) the primary outcome measures include neurological function scores, infarct area or proportion, and all biomarkers indicating IS.

**FIGURE 2:**
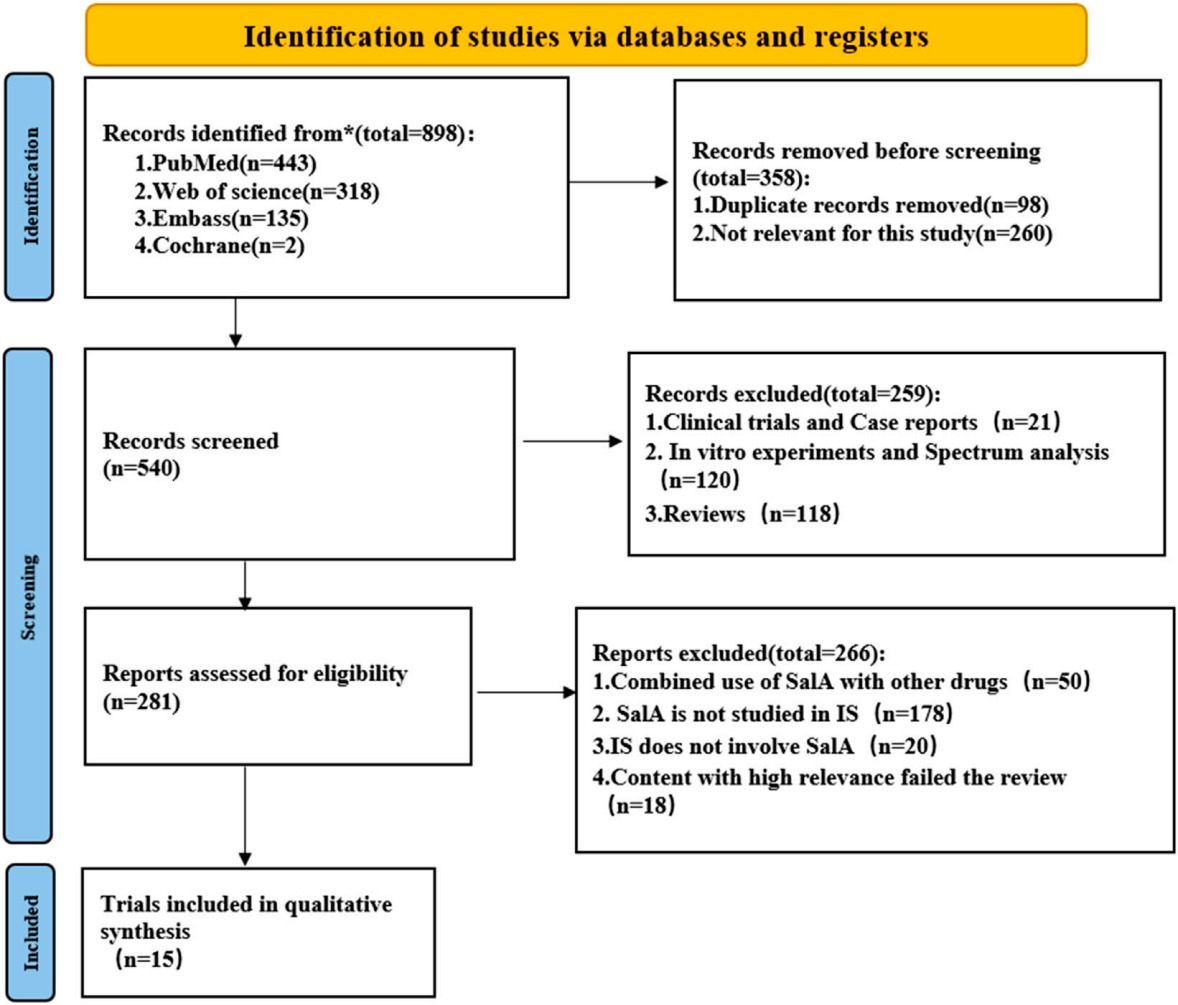
Flowchart of the systematic review process. The paper inclusion process consists of three steps: retrieval, removal of duplicates, and manual screening.

The exclusion criteria for the literature are strictly defined as follows: (1) animal models of non-cerebral ischemia or global cerebral ischemia; (2) reviews, *in vitro* studies, and trials; (3) studies in which SalA was used to intervene in other diseases or was not used at all; (4) studies with insufficient data, including unpublished data; and (5) duplicated studies (Figure 2).

### 2.3 Data extraction

The data extraction table is completed by two independent individuals to ensure accuracy. Additionally, the reference list includes the following information: (1) first author’s name and year of publication; (2) detailed information about the animal model, such as species, gender, and weight; (3) induction method of MCAO and I/R animal models; (4) intervention group: drug used, dosage, and administration method; (5) control group: type, dosage, time, and duration of the nonfunctional substance given; (6) main conclusions of the study; and (7) outcome measures. When animals in the experimental group receive different doses, only the highest dose is recorded. If data are presented in a graphical form, they are measured using digital ruler software. When data are incomplete, we attempt to contact the authors to obtain further information.

### 2.4 Assessment of the risk of bias of included studies

Two research workers independently assessed the quality of the literature using the SYRCLE animal experiment bias risk assessment tool within Review Manager 5.4 software, evaluating 10 criteria: selection bias (sequence generation, baseline characteristics, and allocation concealment), performance bias (random housing and blinding), detection bias (random outcome assessment and blinding), attrition bias (incomplete outcome data), reporting bias (selective outcome reporting), and other potential sources of bias. Each criterion was rated as follows: low risk (method correctly applied), unclear risk (method application unclear), or high risk (method incorrectly applied or not applied). The assessments were carried out independently by both research workers and cross-checked. In case of disagreements, the research workers discussed and reached a consensus; if no consensus was achieved, a third research worker was consulted to make the final decision.

Each item was scored one point if it was met, for a maximum quality score of 10. Discrepancies in scoring were resolved through discussion. We generally consider a score above 6 to be the average score. Importantly, no studies were excluded based on their quality score. The risk-of-bias assessment was instead used to evaluate the overall quality of the evidence base and to inform subsequent sensitivity analyses to investigate potential sources of heterogeneity.

### 2.5 Statistical analyses

Given that the outcome measurements in this study are continuous variables, the standardized mean difference (*SMD*) with a 95% confidence interval (*95% CI*) was used to combine the effect size. When *p* < 0.05, it indicates a significant difference between the intervention and control groups. The heterogeneity evaluation metric used is I-squared (*I* ^*2*^). When *I* ^*2*^ < 50%, it indicates that the studies retrieved are homogeneous, and a fixed-effect model is used to combine the effect size. When *I* ^*2*^ > 50%, it indicates that the quality of the studies retrieved is heterogeneous, and a random-effects model is applied to combine the effect size. To identify potential causes of heterogeneity, subgroup analyses were performed based on animal species, methods, and stages of administration. To assess potential publication bias, the Egger test was used. Additionally, Origin 2021 was used for dose–response interval analysis.

## 3 Results

A total of 898 records were retrieved from four databases using the defined search strategy. After excluding 358 duplicates and irrelevant articles, 540 articles remained. After reviewing the titles or abstracts, 259 studies were excluded, including *in vitro* studies, clinical trials, case reports, and review articles. The remaining 399 articles were read for further screening. A total of 384 studies were excluded for the following reasons: (1) SalA was used in combination with other drugs; (2) non-MACO, MACO/R, or I/R animal models were used; (3) SalA was not used; and (4) high-relevance articles failed the review process. Finally, the remaining 15 papers were included (Figure 2).

### 3.1 Characteristics of included studies

A total of 15 animal experiments from 2016 to 2025 were retrieved, all published in English journals, with a total of 564 animals included: 282 in the intervention group and 282 in the control group. The animal species included SD rats (Feng et al., 2017; Huang and Wang, 2023; Li J. et al., 2025; Ling et al., 2021; Liu et al., 2020; Song et al., 2019; Yang M. Y. et al., 2024; Yang R. et al., 2024; Zhang S. et al., 2022; Zhang W. et al., 2016; Zhang W. et al., 2018; Zhao et al., 2020), Wistar rats (Yang et al., 2022), C57BL/6 mice (Mahmood et al., 2017), and ICR mice (Chien et al., 2016; Yang M. Y. et al., 2024). Anesthesia was induced using pentobarbital sodium (Huang and Wang, 2023; Li J. et al., 2025; Yang et al., 2022), chloral hydrate (Feng et al., 2017; Ling et al., 2021; Liu et al., 2020; Mahmood et al., 2017; Zhang W. et al., 2016; Zhang W. et al., 2018), isoflurane (Feng et al., 2017; Ling et al., 2021; Liu et al., 2020; Mahmood et al., 2017; Zhang W. et al., 2016; W. Zhang et al., 2018), and an unspecified anesthetic (Zhao et al., 2020; Table 1).

**TABLE 1:** Characteristics of the included studies.

Study (year)	Species (sex)	Weight (g)	Animal model	Intervention group (method)	Control group (method)	Main conclusion	Outcome index
Chien M. et al. (2016)	ICR mice (male)	28–30	MCAO model	100 μg/kg (intravenous injection)	No treatment	SalA upregulates BDNF, thereby activating the PI3K/Akt signaling pathway. Additionally, SalA inhibits GSK-3, promoting antiapoptotic and neurogenic pathways. The prevention of p25 generation and Cdk5 activation by SalA confers further benefits in the context of stroke	①
Zhang W. et al. (2016)	SD rats (male)	240–260	MCAO/R model	20 mg/kg SalA	No treatment	SalA exerts protective effects against cerebral ischemia/reperfusion injury by activating the Nrf2/HO-1 signaling pathway. It enhances Nrf2 synthesis and nuclear translocation, thereby promoting the expression of the downstream antioxidant protein HO-1	①②③
Feng S. et al. (2017)	SD rats (male)	180–200	tMCAO model	10 mg/kg SalA (intravenous injection)	No treatment	SalA exerts a greater regulatory effect on serum metabolic disturbances than on tissue metabolic alterations induced by ischemia/reperfusion injury	①②③④⑤⑥
Mahmood. et al. (2017)	C57BL/6 mice (male)	20–28	tMCAO model	5 mg/kg (intragastric gavage)	Treated with saline	SalA can inhibit the effects of eNOS uncoupling-dependent nitrosative stress and calpain signaling during cerebral ischemia, thereby improving glucose metabolism during ischemic events	①②⑬
Zhang W. et al. (2018)	SD rats (male)	240–260	MCAO model	20 mg/kg (intravenous injection)	Administer equal volume of normal saline	SalA exhibits significant neuroprotective effects against I/R injury. It protects the blood–brain barrier and exerts anti-inflammatory actions by suppressing the I/R-induced expression of MMP-9 in rats and inhibiting the activation of NF-κB	①②③④⑤⑥⑪⑫
Song J. et al. (2019)	SD rats (male)	240–260	MCAO/R model	20 mg/kg (intravenous injection)	NA	This is the first report to demonstrate that SalA, in part via the AKT/FOXO3a/BIM pathway, attenuates cerebral I/R injury, thereby offering a new and promising strategy for neuroprotection	①②⑬⑭
Zhao J. et al. (2020)	SD rats (male)	250–300	MCAO model	20 mg/kg (intraperitoneal injection)	No treatment	SalA alleviates brain damage, inflammation, and apoptosis induced by cerebral ischemia/reperfusion injury in rats by modulating the miR-499a/DDK1 pathway	①②③④⑤⑥⑦ ⑧⑩
Ling Y. et al. (2021)	SD rats (male)	240–260	I/R model	10 mg/kg (tail vein injection)	1 mg/kg normal saline	LR2 and TLR4 are potential targets of SalA, which plays a key role in ameliorating microglial inflammation through a MyD88-mediated pathway	①③④⑥
Liu C. et al. (2020)	SD rats (male)	220–260	Autologous thrombotic stroke model	10 mg/kg (intragastric gavage)	0.5% CMC-Na, ig	SalA prevents cerebral vascular endothelial injury induced by acute ischemic stroke by inhibiting the src signaling pathway, suggesting that pretreatment with SalA represents a potential therapeutic strategy for preventing ischemic stroke	②⑪⑫
Yang Y. et al. (2022)	Wistar rat	240–260	CCI (two-vessel occlusion) model	20 mg/kg	Treated with saline	SalA treatment alleviates cognitive impairment in rats induced by CCI, potentially by reducing inflammation and apoptosis through modulation of the neuronal drd2/Cryab/NF-κB pathway	⑩
Zhang S. et al. (2022)	SD rats (male)	230–260	Electrocoagulation-induced autologous thrombotic stroke model	10 mg/kg (intragastric gavage)	NA	Chronic administration of SalA accelerates neural recovery by promoting endogenous neurogenesis and axonal sprouting, as well as by inhibiting neuronal apoptosis after ischemic stroke via the Wnt3a/β-catenin signaling pathway. This contributes to improved long-term outcomes in stroke	①②⑨⑩
Huang S. et al. (2023)	SD rats (male)	250–350	MCAO model	20 mg/kg (intravenous injection)	Intraperitoneally injected with 1 mL/kg normal saline	SalA modulates the mir-212-3p/SOX7 axis to activate the Wnt/β-catenin pathway, thereby ameliorating brain injury, edema, and neurological deficits induced by cerebral ischemia/reperfusion injury (CIRI), and providing a novel therapeutic approach and strategy for ischemic brain injury	②③④⑤⑥
Yang M. Y. et al. (2024)	ICR mice/SD rats (male)	16–18/230–240	tMCAO model	20 mg/kg (intravenous injection)	Same volumes of blank liposome solutions were administered in the same manner	Liposome/SalA exhibits significant neuroprotective effects and demonstrates excellent biosafety	①③⑤⑭
Yang R. et al. (2024)	SD rats (male)	280–320	tMCAO model	8 mg/kg (intravenous injection)	Same volume of normal saline	SalA mitigates cerebral infarction, brain edema, and brain atrophy in rats with cerebral ischemia/reperfusion injury (CIRI), reducing both neurological deficits and histopathological damage by inhibiting neuronal apoptosis through modulation of the PKA/creb/c-Fos signaling pathway	①⑦⑧⑨
Li J. et al. (2025)	SD rats (male)	280–320	Photothrombotic model	2 mg/kg (tail vein injection)	100 g/0.1 mL normal saline	SalA exerts neuroprotective effects in stroke models by inducing synaptic suppression, thereby reducing acute-phase infarction and promoting recovery during the later stages of AIS	①

①, Cerebral infarction area; ②, NDS; ③, cerebral edema volume; ④, TNF-α; ⑤, IL-6; ⑥, IL-1β; ⑦, Bax/β-actin; ⑧, Bcl-2/β-actin; ⑨, Bcl-2/Bax; ⑩, Caspase3; ⑪, ZO-1/β-actin; ⑫, occludin/β-actin; ⑬, p-Akt/Akt; ⑭, the ratio of NeuN and TUNEL, expression in cells.

All animal models used in this study were described according to the inclusion and exclusion criteria from the literature, reflecting the pathological process of IS. The intervention group was treated with SalA monotherapy. The control group received an equal volume of physiological saline, drug carrier treatment, or no treatment. In evaluating the effect of SalA on IS, 12 studies reported cerebral infarction volume, nine studies reported NODs, seven studies reported brain edema volume, five studies reported tumor necrosis factor-a (TNF-α), five studies reported interleukin 6 (IL-6), five studies reported IL-1β, three studies reported Caspase-3, two studies reported Bcl-2-associated X protein (Bax)/β-actin, two studies reported B-cell lymphoma-2 (Bcl-2)/β-actin, two studies reported Bcl-2/Bax, two studies reported the expression of Caspase-3 protein, two studies reported ZO-1/β-actin, two studies reported occludin/β-actin, two studies reported p-AKT/AKT, and two studies reported the positive expression rates of neuronal nuclei antigen (NeuN) and terminal urine nucleotide extraction ligation (TUNEL) in cells (Table 1).

### 3.2 Characteristics of the included studies

### 3.3 Research quality

**FIGURE 3:**
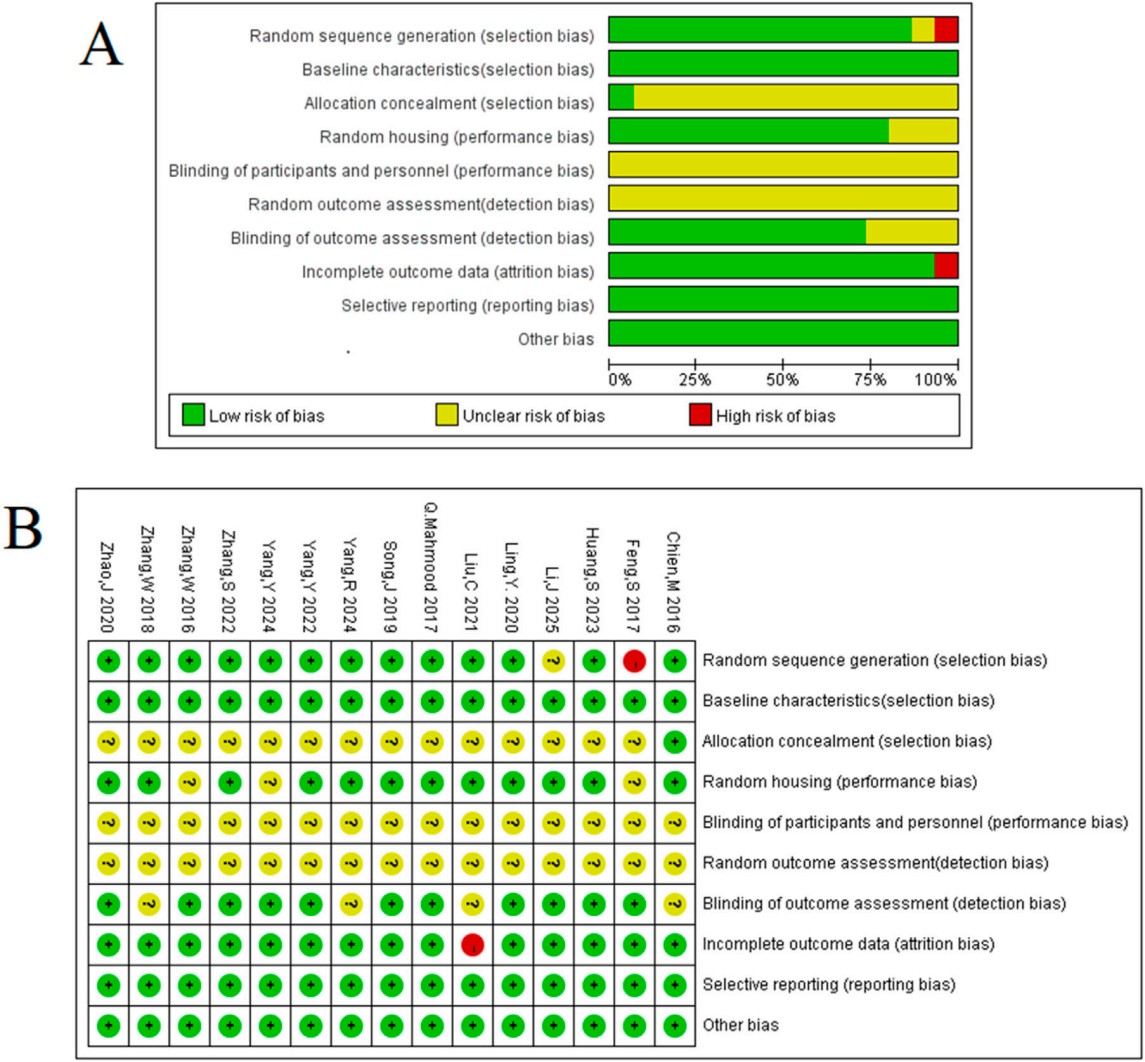
Methodologic quality assessment of the risk of bias: **(A)** risk-of-bias graph: review authors’ judgements about each risk-of-bias item presented as percentages across all included studies. **(B)** Risk-of-bias summary: review authors’ judgements about each risk-of-bias item for each included study.

The methodological quality of the 15 included studies was evaluated using the SYRCLE animal experiment bias risk assessment tool to identify both strengths and areas for improvement in the current body of preclinical literature. The results, summarized in Figure 3, indicate a consistent adherence to certain reporting standards, with total quality scores for the studies ranging from 5 to 7 out of a possible 10.

Encouragingly, all included studies were published in peer-reviewed journals and reported compliance with animal welfare regulations, establishing a foundational level of scientific rigor. Moreover, a majority of experiments demonstrated good laboratory practice by reporting temperature control (80%) and using anesthetics without significant intrinsic neuroprotective activity (87%). These aspects enhance the reliability of the reported findings. However, the assessment also highlighted several opportunities for methodological enhancement in future preclinical research. Key areas such as sample size calculation, allocation concealment, and blinded assessment of outcomes were not consistently reported across the studies (0%, 0%, and 27%, respectively).

Although these methodological gaps are common in exploratory preclinical research and represent a known challenge in the field, they also underscore the importance of a systematic review. By synthesizing data from these studies, our meta-analysis provides a comprehensive overview of the current evidence landscape. The identified risks of bias, particularly in selection and detection, are considered significant potential sources of the statistical heterogeneity observed. Acknowledging these limitations allows for a more nuanced interpretation of the pooled results and provides clear, actionable recommendations for improving the design and reporting of future animal studies in this area. Therefore, the current body of evidence was deemed suitable for a meta-analysis aimed at summarizing existing findings and guiding future, more robust research.

### 3.4 Effectiveness

#### 3.4.1 Pathology and validity of basic indicators

To quantitatively evaluate the overall effectiveness of SalA in preclinical models of ischemic stroke, we conducted a meta-analysis on the three primary efficacy outcomes: cerebral infarct volume, neurological deficit score (NDS), and brain edema.

The pooled results demonstrate a significant neuroprotective effect of SalA compared to the control group. The analysis of 12 studies (n = 223 animals) revealed that SalA treatment significantly reduced cerebral infarct volume (*SMD* = −4.67, 95% *CI* = [−5.98, −3.36], and p < 0.001). Similarly, data from nine studies (n = 164) showed a significant improvement in neurological function, with SalA treatment leading to lower deficit scores (*SMD* = −6.39, 95% *CI* = [−9.091, −3.688], and *p* < 0.001). Furthermore, the analysis of seven studies (n = 88) confirmed that SalA markedly alleviated brain edema (*SMD* = −5.291, 95% *CI* = [−7.607, −2.975], and *p* < 0.001). These findings provide robust quantitative evidence for the therapeutic potential of SalA in mitigating key aspects of ischemic brain injury.

## 4 Correlation analysis between different variables

**FIGURE 4:**
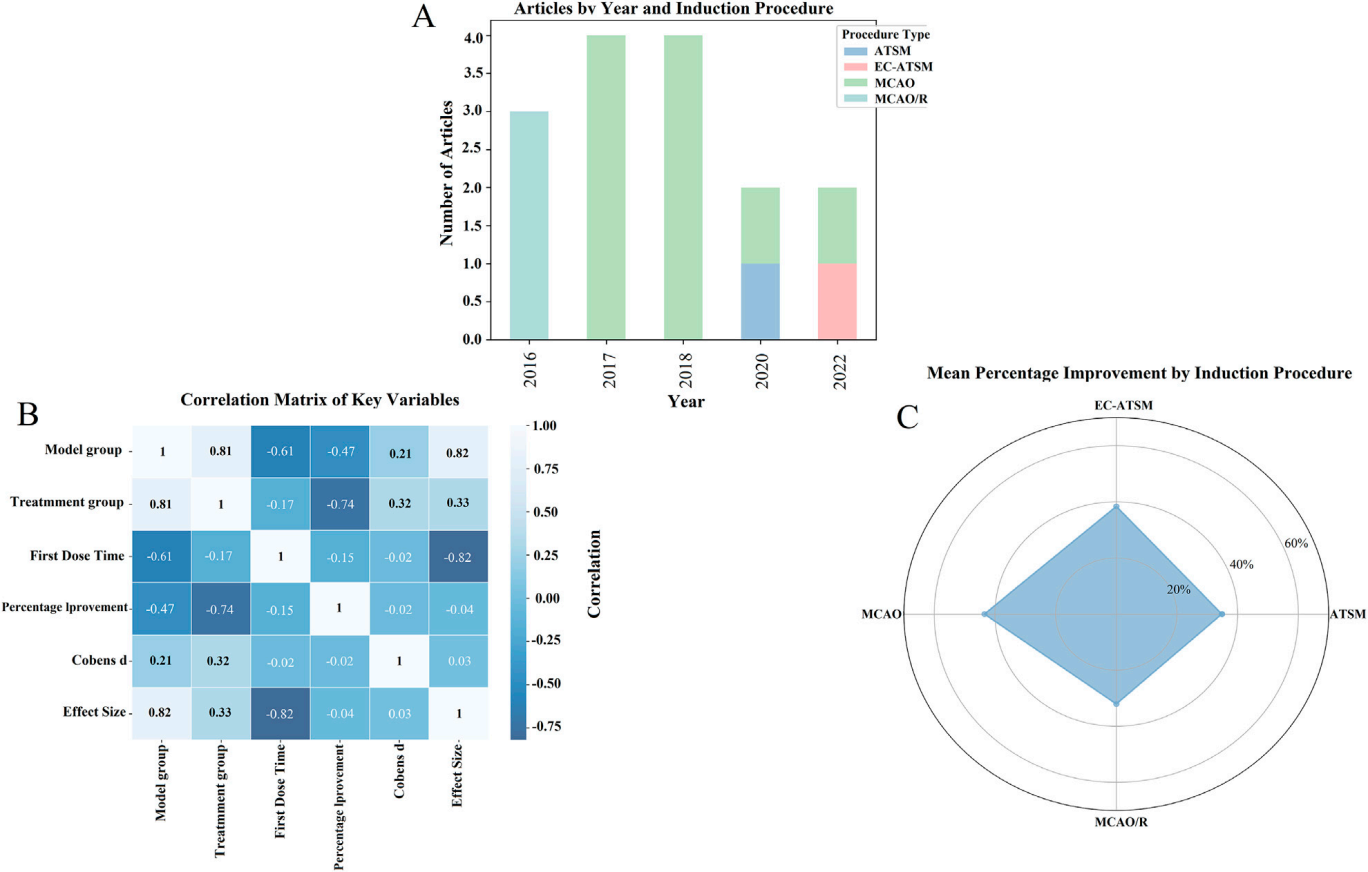
Impact of different induction protocols and key variables on drug improvement effects: an exploration based on meta-analysis. **(A)** Number of articles under different years and induction protocols; **(B)** correlation matrix of key variables; **(C)** average percentage improvement under different induction protocols.

To explore the potential sources of heterogeneity and generate hypotheses for future research, we conducted a series of exploratory analyses based on study characteristics, as depicted in Figure 4. It is critical to note that due to the limited number of included studies, these analyses are considered hypothesis-generating rather than confirmatory, and the observed trends require validation in future, more rigorous studies.

Figure 4A illustrates the distribution of included studies based on the publication year and the type of the ischemia model used. This visualization highlights a potential shift in model preference over time, with MCAO and MCAO/R models being more common in earlier studies, whereas newer models including ATSM and EC-ATSM have been utilized more recently. This observation may reflect an evolving focus in the field toward models with different pathophysiological features.

Furthermore, we explored the relationships between key study variables using a correlation matrix, as shown in Figure 4B. Strong correlations were observed between the treatment and model groups (*r* = 0.81), and a strong negative correlation was noted between the time of first drug administration and the effect size (*r* = −0.82), suggesting that earlier administration may be associated with better outcomes. However, these correlation coefficients are presented for descriptive purposes and were not tested for statistical significance due to the small sample size (n = 15 studies), which limits the ability to draw firm conclusions.

Figure 4C provides a descriptive summary of the average percentage improvement in outcomes for each model type via a radar plot. Although these raw data might suggest different magnitudes of SalA’s effect across models, this observation is based on a very small number of studies for each model and should be interpreted with extreme caution.

In summary, these exploratory analyses suggest that variables such as the choice of animal model and the timing of administration may influence the therapeutic effect of SalA. These findings should not be viewed as conclusive evidence but rather as preliminary insights that highlight critical areas for future investigation. In particular, future preclinical studies should aim to directly compare different dosages and administration windows in standardized models to optimize the therapeutic protocol for SalA.

## 5 Exploration of dose–effect and safety considerations

**FIGURE 5:**
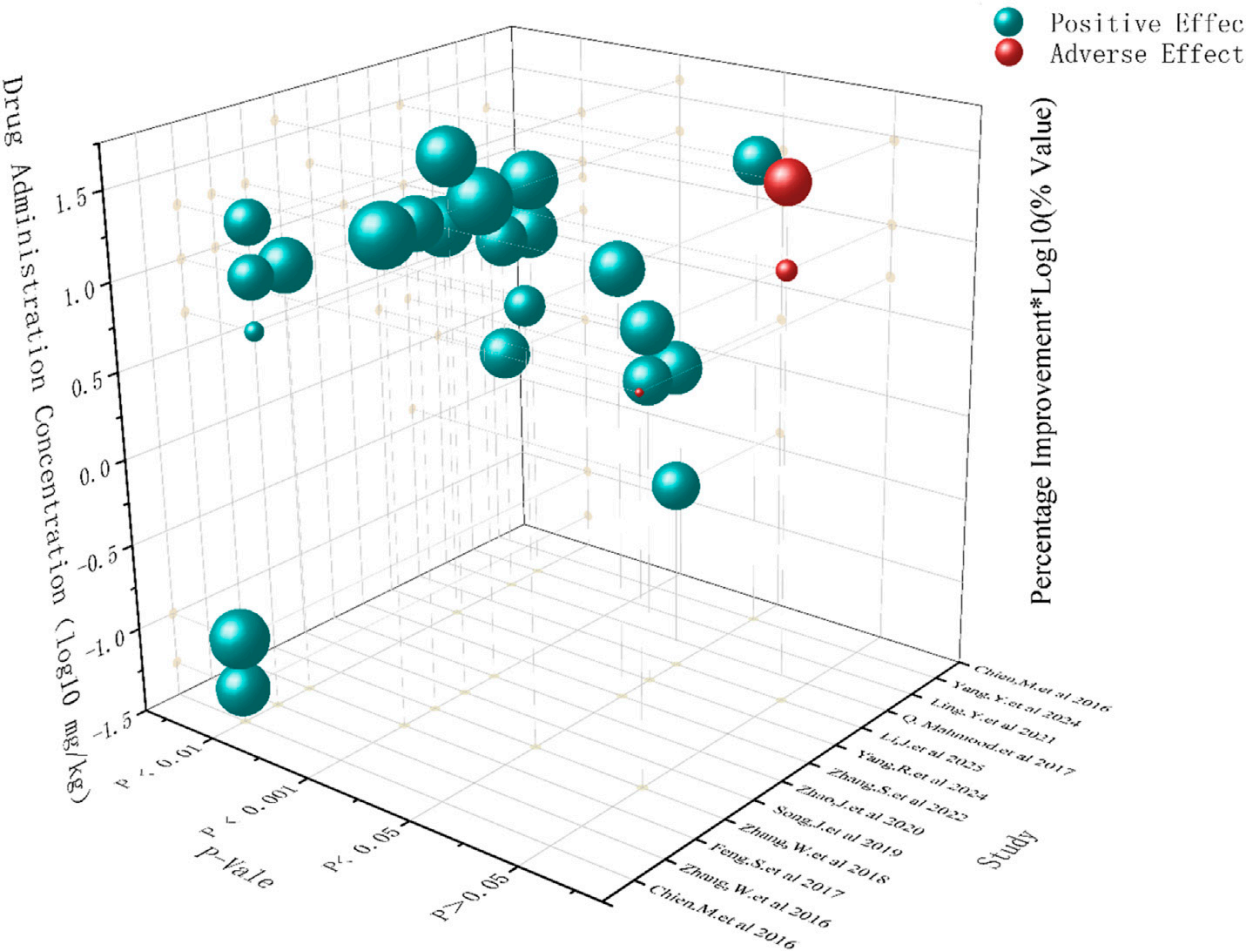
Three-dimensional effect analysis based on drug concentration and improvement effects.

To investigate the potential influence of dosage on the efficacy of SalA, we examined the effect sizes reported across different dose ranges in the included studies. It is important to preface this analysis by stating that due to the limited number of distinct dose groups and the variability in experimental designs, a formal dose–response meta-regression analysis could not be performed. Therefore, the following discussion is based on a qualitative assessment of the available data and should be considered exploratory (Figure 5).

A qualitative review of the data suggests a potential positive association between SalA concentration and treatment efficacy. Studies using higher doses (e.g., 20 mg/kg or more) generally appeared to yield larger effect sizes than those using lower doses (e.g., <10 mg/kg). This observation is supported by the subgroup analysis for neurological deficit scores, presented in Figure 7B, where the 20 mg/kg subgroup demonstrated a pronounced therapeutic effect. This dose level may represent a promising range for achieving significant efficacy and warrants prioritization in future preclinical and clinical investigations.

Regarding the balance between efficacy and safety, the reporting of adverse events across the included studies was sparse and non-systematic, precluding a formal meta-analysis of safety outcomes. Although most studies did not report observable adverse effects, one study noted potential adverse outcomes at a high dose (20 mg/kg). This highlights a critical knowledge gap. Although higher doses appear to be more effective, it is essential to systematically evaluate the safety profile of SalA across a range of doses. Future studies should be designed with *a priori* definitions of potential adverse events and include rigorous safety monitoring to establish a therapeutic window that optimally balances efficacy and safety.

## 6 Results of meta-analyses

### 6.1 Study selection and characteristics

The literature search initially identified 898 records. After removing duplicates and screening titles and abstracts, 281 full-text articles were assessed for eligibility. This process resulted in the final inclusion of 15 preclinical studies that met all criteria (Figure 2). The key characteristics of these 15 studies, which involved a total of 564 animals, are summarized in Table 1. The methodological quality assessment, as detailed in Figure 3, revealed that although all studies adhered to certain reporting standards, significant risks of bias were prevalent, particularly in the domains of sample size calculation, allocation concealment, and blinded outcome assessment, which are critical for the interpretation of the following results.

### 6.2 Effects on primary brain injury outcomes

The meta-analysis demonstrated that SalA treatment led to significant improvements in the three primary measures of ischemic brain injury. The main findings of the meta-analyses for all outcomes are summarized in Table 2.

**TABLE 2:** Summary of findings for the efficacy of salvianolic acid A in preclinical models of ischemic stroke.

Outcome	No. of studies	Total animals (N)	Pooled effect size (SMD [95% *CI*])	Heterogeneity (*I* ^*2*^)	Risk of bias	Certainty of evidence
Primary brain injury outcome						
Cerebral infarct volume	12	223	−4.67 [−5.98, −3.36]	85.50%	High	Low
Neurological deficit score	9	164	−6.39 [−9.091, −3.688]	66.80%	High	Low
Brain edema	7	88	−5.291 [−7.607, −2.975]	83.50%	High	Low
Inflammatory marker						
TNF-α	5	70	−4.14 [−6.52, −1.77]	85.80%	High	Very low
IL-6	5	56	−5.72 [−8.71, −1.38]	89.70%	High	Very low
IL-1β	5	70	−4.77 [−6.75, −2.79]	72.30%	High	Low
Apoptosis marker						
Bcl-2/Bax ratio	2	18	6.532 [3.93, 9.13]	0%	Moderate	Moderate
Caspase-3	3	30	−3.495 [−4.60, −2.39]	0%	Moderate	Moderate
Blood–brain barrier marker						
ZO-1	2	24	4.97 [3.20, 6.74]	36.50%	Moderate	Low
Occludin	2	24	1.45 [−0.39, 3.34]	72.60%	Moderate	Low

**FIGURE 6:**
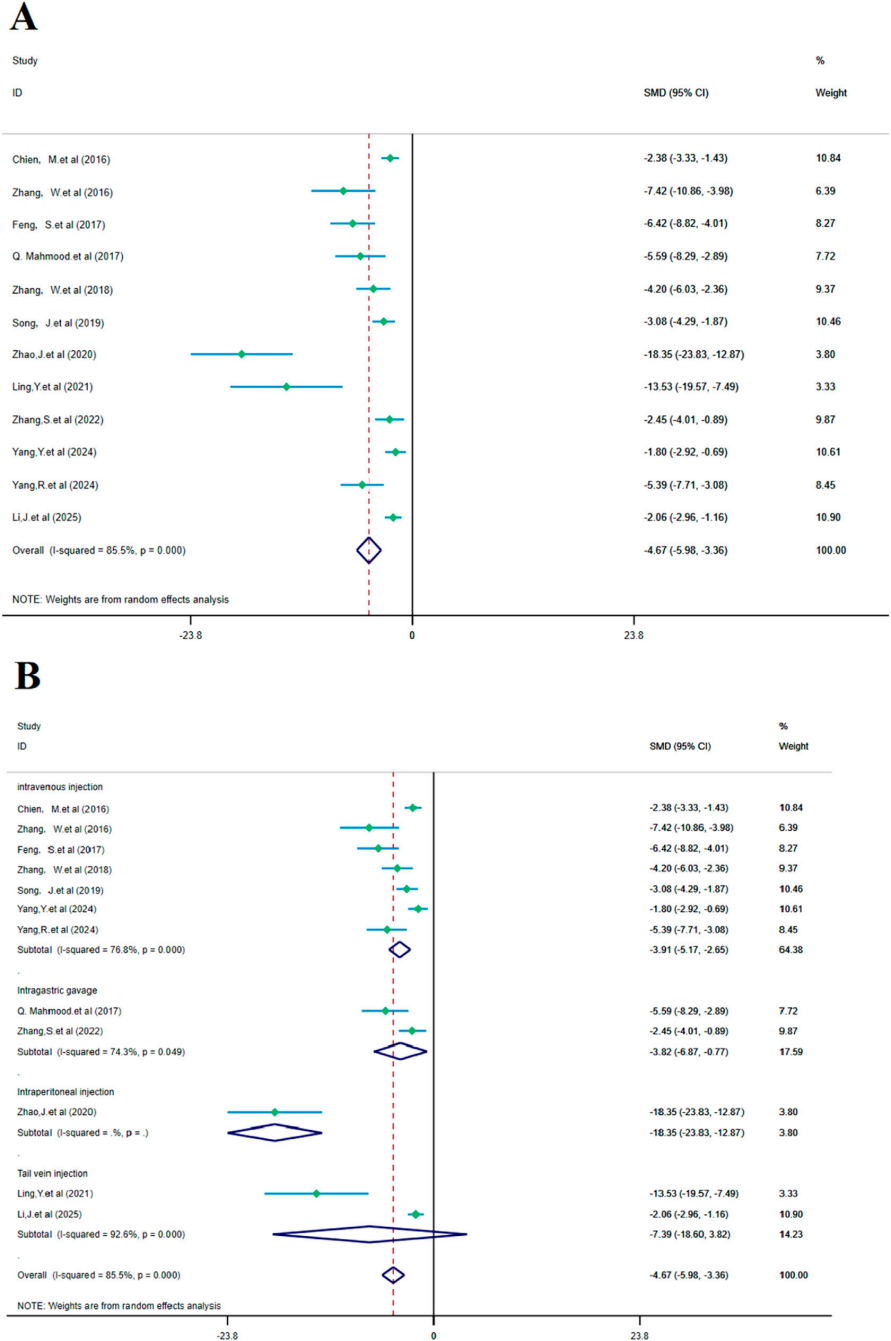
Forest plot (effect size and 95% CI) summarizes the comparison of SalA with the control group on cerebral infarction area and its subgroup analysis: **(A)** cerebral infarction area and **(B)** subgroup analysis.

The analysis of 12 studies confirmed that SalA positively reduced the infarct area in IS compared to the control group [*SMD* = −4.67, *95% CI* = (−5.98, −3.36), *p* < 0.00, n = 223, and n (treatment/model) = 112/111]. Due to significant heterogeneity between studies (*I* ^*2*^ = 87.8 and *p* < 0.001), the heterogeneity for this analysis was *I* ^*2*^ = 85.5%, and a subgroup analysis based on the administration method was performed (Figure 6A).

Subgroup analysis based on the administration method showed a positive reduction in heterogeneity. In the intravenous injection subgroup, heterogeneity remained high (*I* ^*2*^ = 76.8% and *p* < 0.001), but SalA still showed positive effects in the intravenous injection group [*SMD* = −3.91, *95% CI* = (−5.17, −2.65), *p* < 0.001, and n = 130]. In the gastric gavage subgroup, heterogeneity decreased (*I* ^*2*^ = 74.3% and *p* < 0.05), and SalA showed positive effects with this administration method [*SMD* = −3.82, *95% CI* = (−6.87, −0.77), *p* < 0.001, and n = 24]. In the tail vein injection subgroup, n = 20/22 (treatment/model), heterogeneity was high (*I* ^*2*^ = 92.6% and *p* < 0.001), but SalA still showed positive effects with this administration method [*SMD* = −7.39, *95% CI* = (−18.60, 3.82), *p* < 0.001, and n = 42]. As only one study was included, heterogeneity was not tested in the intraperitoneal injection group [*SMD* = −18.35, *95% CI* = (−23.83, −12.87), *p* < 0.001, and n = 24]. Despite high heterogeneity in the overall analysis, the effects of SalA were particularly positive in the intravenous and tail vein injection subgroups, suggesting that different administration methods may have an impact on the therapeutic effects (Figure 6B).

**FIGURE 7:**
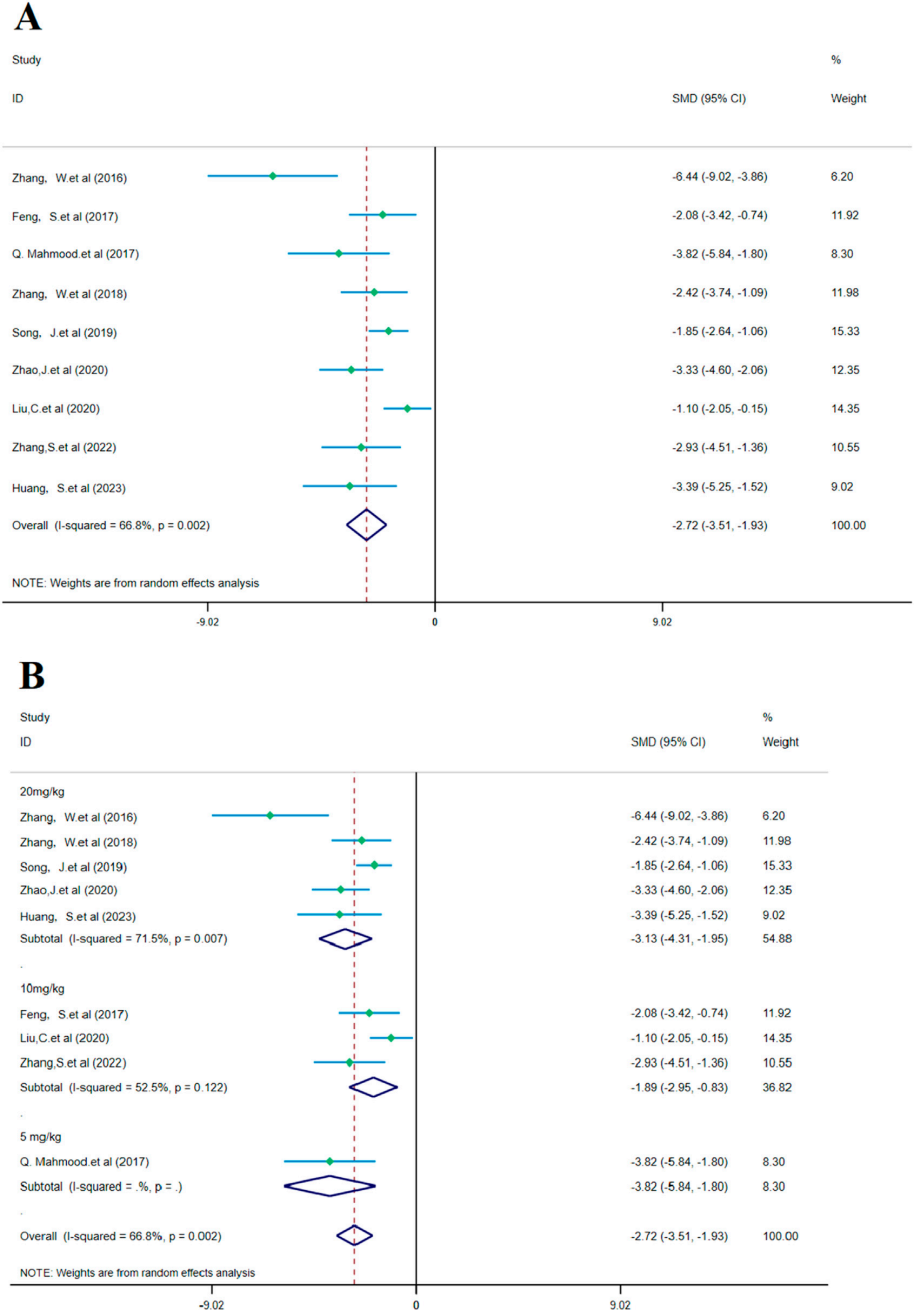
Forest plot (effect size and 95% CI) summarizes the comparison of SalA with the control group on NDS and its subgroup analysis: **(A)** NDS and **(B)** subgroup analysis.

According to the meta-analysis of nine studies, SalA showed positive effects in alleviating the neurological deficit scores in IS. Compared to the control group, SalA reduced the neurological deficit scores [*SMD* = −6.39, *95% CI* = (−9.091, −3.688), *p* < 0.001, and n = 164]. However, due to significant heterogeneity between studies (*I* ^*2*^ = 66.8% and *p* < 0.001), we performed a subgroup analysis based on different drug concentrations (20 mg/kg, 10 mg/kg, and 5 mg/kg) (Figure 7A).

Subgroup analysis based on drug concentrations: 20 mg/kg subgroup: this group had high heterogeneity (*I* ^*2*^ = 71.5% and *p* = 0.007), showing considerable variability between studies. SalA positively reduced the neurological deficit scores at the concentration of 20 mg/kg [SMD = −3.130, 95% CI = (−4.307, −1.954), p < 0.001, and n = 82]. This subgroup accounted for 54.88% of the total analysis; 10 mg/kg subgroup: this group had lower heterogeneity (I2 = 52.5% and p = 0.122). SalA also positively reduced the neurological deficit scores at the 10 mg/kg concentration [SMD = −1.893, 95% CI = (−2.952, −0.835), p < 0.001, and n = 50]. This subgroup accounted for 36.82% of the total analysis; 5 mg/kg subgroup: only one study was included in this group, with zero heterogeneity (*I* ^*2*^ = 0%), indicating almost no variability. However, SalA still significantly reduced the neurological deficit scores [*SMD* = −3.823, *95% CI* = (−5.844, −1.801), *p* < 0.001, and n = 32] (Figure 7B). The results of this meta-analysis show that SalA significantly improved the neurological deficit scores in IS models across different concentration groups, and the overall effect was statistically positive. Heterogeneity varied at different concentrations, with the 20 mg/kg group showing considerable variation between studies. Future research may need to further explore the relationship between drug concentration and therapeutic effects, in particular, the effects and heterogeneity at higher concentrations.

**FIGURE 8:**
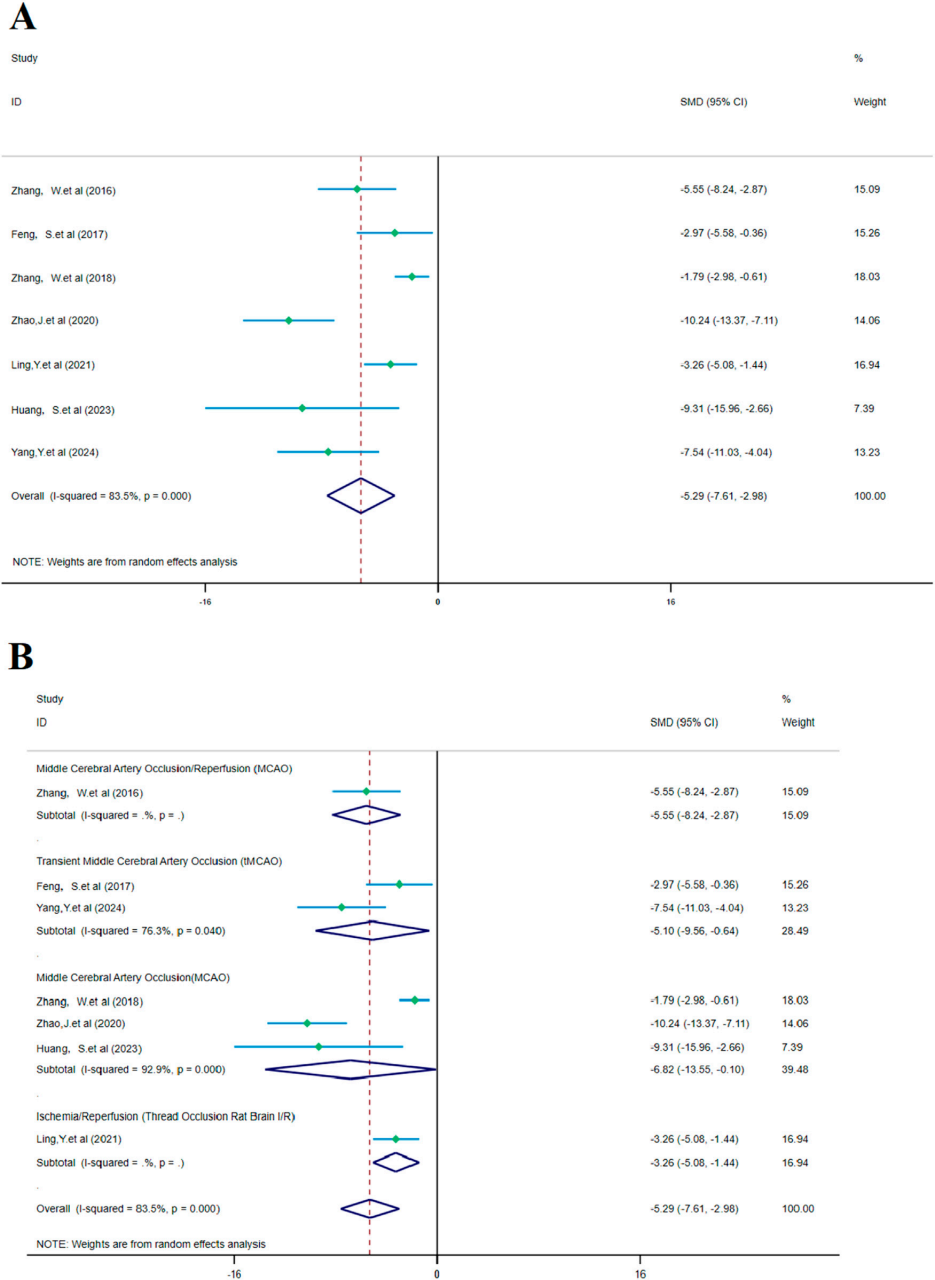
Forest plot (effect size and 95% confidence interval) summarizes the comparison between SalA and the control group for brain edema volume and its subgroup analysis: **(A)** brain edema volume and **(B)** subgroup analysis.

The analysis of seven studies showed that SalA had a positive effect in reducing the brain edema area after IS injury compared to the control group [*SMD =* −5.291, *95% CI* (−7.607, −2.975), *p* < 0.001, and n = 88]. Due to significant heterogeneity between groups (*I* ^*2*^ = 83.5% and *p* < 0.001) (Figure 8A), we performed a subgroup analysis based on animal models for the seven studies. The results showed that SalA positively reduced the brain edema area in all model groups [MACO group: *SMD* = −6.82, *95% CI* = (−13.55, −0.10), *p* < 0.001, and n = 46 (weight = 39.48%); tMACO group: *SMD* = −5.1, *95% CI* = (−9.56, −0.64), *p* < 0.001, and n = 18 (weight = 28.49%)]. The I/R group and MACO/R group each included only one study, with zero heterogeneity, but still showed positive therapeutic effects [MACO/R group: *SMD* = −5.54, *95% CI* = (−8.23, −2.87), *p* < 0.001, and n = 12 (weight = 15.09%); I/R group: *SMD* = −3.26, *95% CI* = (−5.08, −1.44), *p* < 0.001, and n = 12 (weight = 16.94%)] (Figure 8B).

### 6.3 Modulatory effects on markers of inflammation

SalA demonstrated a strong anti-inflammatory effect by significantly reducing the levels of key pro-inflammatory cytokines. The pooled analysis of five studies showed a marked reduction in TNF-α (n = 70; *SMD* = −4.14 and 95% *CI* = [−6.52, −1.77]; Supplementary Figure S1) and IL-6 (n = 56; *SMD* = −5.72 and 95% *CI* = [−8.71, −1.38]; Supplementary Figure S2). Similarly, an analysis of another five studies confirmed a significant decrease in IL-1β levels (n = 70; *SMD* = −4.77 and 95% *CI* [−6.75, −2.79]; Supplementary Figure S3). All these analyses exhibited very high heterogeneity (*I* ^*2*^ > 70%), likely stemming from variations in the timing of sample collection and the specific assay methods used across studies.

### 6.4 Regulatory effects on apoptosis pathways

The meta-analysis of apoptosis-related proteins indicated a consistent and significant antiapoptotic effect of SalA. The pooled data from two studies (n = 18) showed that SalA treatment significantly upregulated the Bcl-2/Bax ratio (*SMD* = 6.532 and 95% *CI* = [3.93, 9.13]; Supplementary Figure S6). Akt, as a key component of the antiapoptotic pathway, is influenced by the therapeutic effects of SalA, which affect the ratio of p-Akt/Akt. In two studies, the following data were found: n = 20; *SMD* = 3.447, 95% *CI* = [1.96, 4.93], and *p* < 0.001 (Supplementary Figure S7). Complementing this finding, data from three studies (n = 30) revealed a significant reduction in Caspase-3 levels (*SMD* = −3.495 and 95% *CI* = [−4.60, −2.39]; Supplementary Figure S8). Notably, both of these analyses exhibited low-to-no heterogeneity (*I* ^*2*^ = 0%), suggesting a more robust and consistent effect of SalA on these specific molecular targets.

### 6.5 Effects on BBB integrity

The analysis of tight junction proteins, key indicators of BBB integrity, showed that SalA had a positive and significant effect on ZO-1 expression based on two studies (n = 24; *SMD* = 4.97 and 95% *CI* = [3.20, 6.74]; *I* ^*2*^ = 36.5%; Supplementary Figure S9). In contrast, for occludin, the pooled effect from two studies was not statistically significant (*SMD* = 1.45 and 95% *CI* = [−0.39, 3.34]; *I* ^*2*^ = 72.6%; Supplementary Figure S10).

### 6.6 Publication bias assessment

**FIGURE 9:**
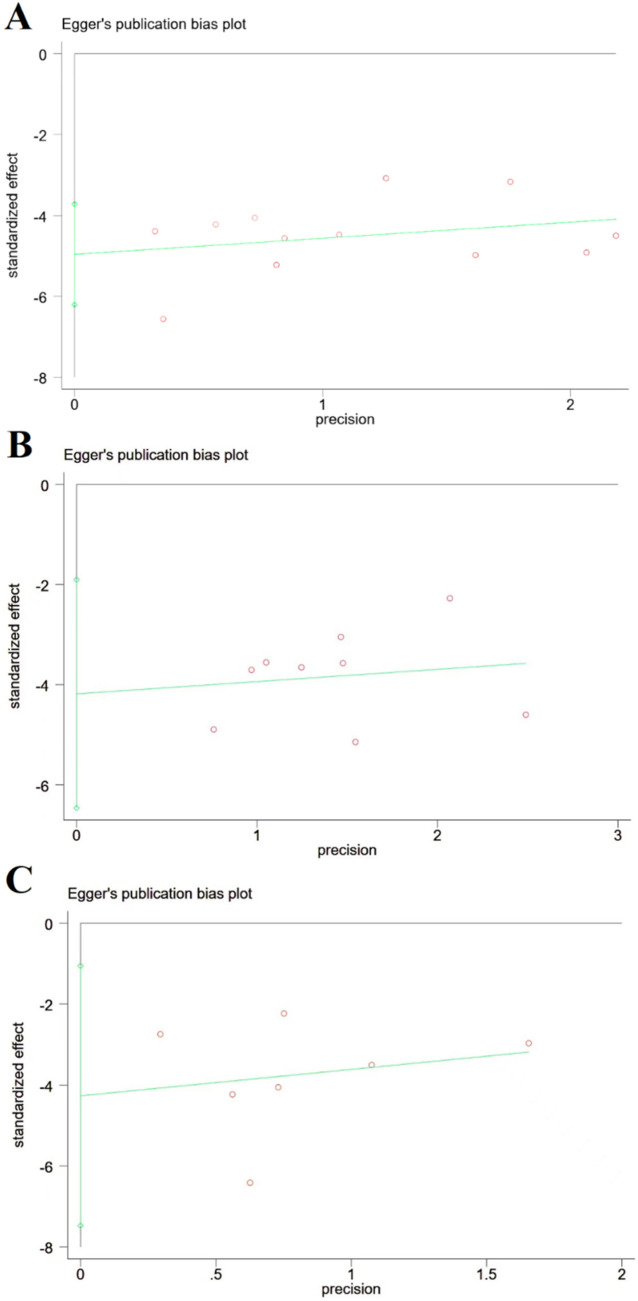
Publication bias based on cerebral infarction area, NDS, and brain edema volume. **(A)** Cerebral infarction area; **(B)** NDS; and **(C)** brain edema volume, and there is no publication bias for cerebral infarction area, NDS, and brain edema volume.

Publication bias for the three primary outcomes was visually inspected using funnel plots (Figures 9A–C) and formally tested with Egger’s regression test. No significant publication bias was detected for cerebral infarct volume (*t* = −8.88 and *p* > 0.05), NDS (*t* = −4.3 and *p* > 0.05), or brain edema (*t* = −3.42 and *p* > 0.05); however, the power of these tests is limited by the small number of studies in each analysis.

### 6.7 Sensitivity analysis

To evaluate the stability of the primary outcome measures (including cerebral infarction area, NDS, and cerebral edema volume), a sensitivity analysis was conducted. The analysis showed no significant differences in the results, indicating the robustness of the study findings (Supplementary Figure S11).

## 7 Discussion

### 7.1 Principal findings and evidence synthesis

To our knowledge, this is the first systematic review and meta-analysis to quantitatively synthesize the preclinical evidence for SalA in treating ischemic stroke. Our pooled results demonstrate that SalA administration in animal models leads to a statistically significant reduction in cerebral infarct volume, neurological deficits, and brain edema. Furthermore, our analyses provide quantitative support for the multi-target nature of SalA, showing consistent modulatory effects on key pathways of inflammation, apoptosis, and blood–brain barrier integrity. However, these promising findings must be interpreted with considerable caution. The certainty of this evidence is significantly limited by the high risk of bias identified across the included studies—particularly, the universal lack of sample size calculation and blinding—and the substantial statistical heterogeneity observed in most of our primary analyses.

### 7.2 Interpretation of mechanistic findings

Our meta-analysis confirmed that SalA’s neuroprotective effects are associated with potent anti-inflammatory and antiapoptotic activities. The significant reduction in pro-inflammatory cytokines such as TNF-α, IL-6, and IL-1β (Supplementary Figures S1-S3) provides robust evidence for its anti-inflammatory role. Several included studies attributed this effect to the inhibition of pathways, including NF-κB (Zhang et al., 2018). Similarly, the strong and consistent effect on increasing the Bcl-2/Bax ratio and decreasing Caspase-3 levels (Supplementary Figures S6-S8), which notably showed low heterogeneity, underscores a reliable antiapoptotic mechanism. These findings suggest that SalA may act by rebalancing the cellular response to ischemic stress, shifting it away from cell death and toward survival. The positive effect on the tight junction protein ZO-1 (Supplementary Figure S9) further suggests that SalA contributes to the restoration of blood–brain barrier integrity, a critical step in mitigating secondary injury.

**FIGURE 10:**
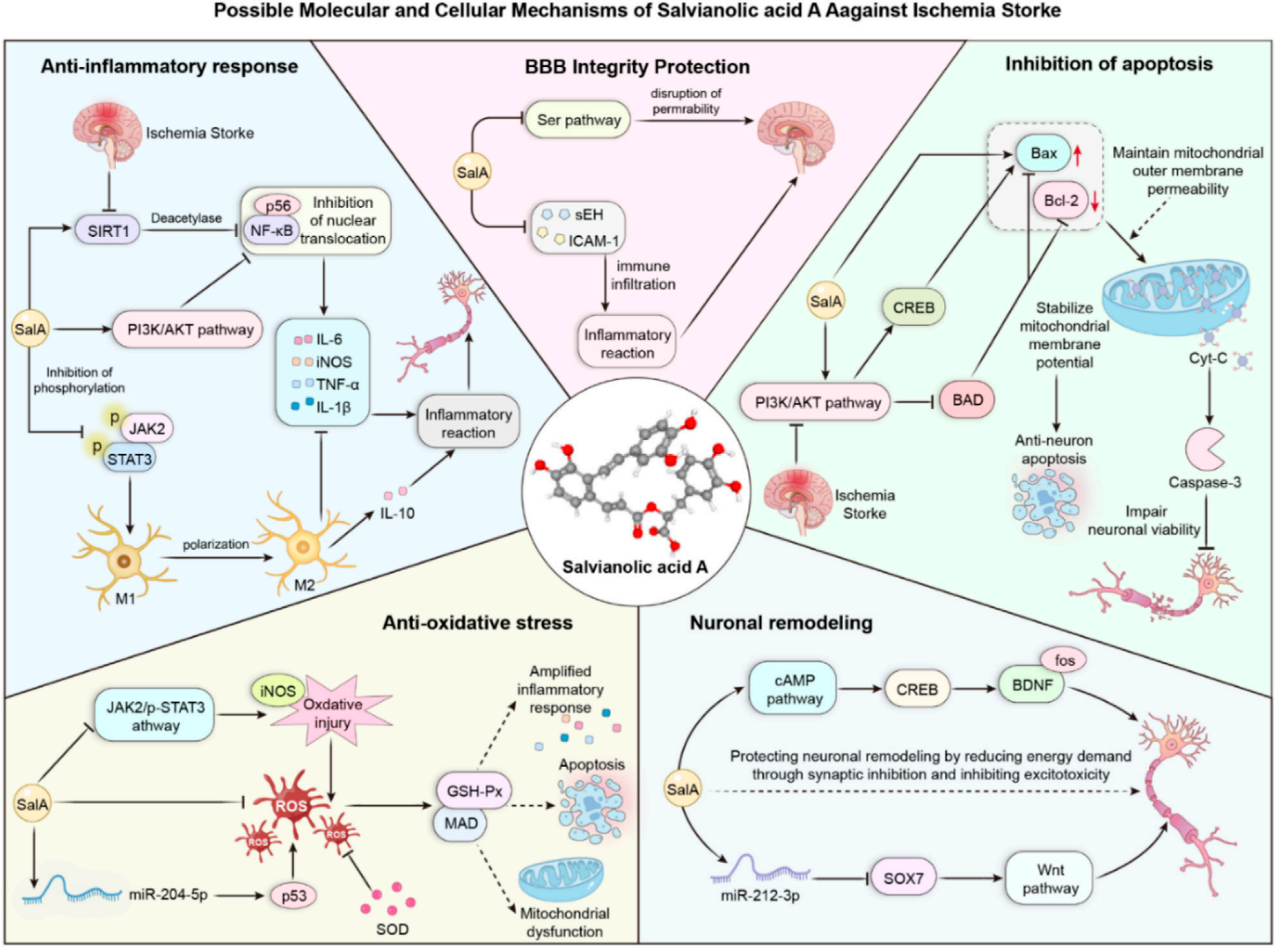
Potential mechanism of SalA in protecting against ischemic stroke.

Taken together, our analysis of molecular markers suggests that SalA exerts its neuroprotective effects through a multi-pronged approach, targeting key pathological cascades in ischemic stroke. We have integrated these findings into a schematic diagram that illustrates the potential interconnected mechanisms of action, highlighting SalA’s role in modulating inflammation, apoptosis, and blood–brain barrier stability (Figure 10).

### 7.3 Methodological quality and heterogeneity

A critical finding of this review is the overall moderate-to-low methodological quality of the current preclinical evidence base, as detailed in our risk-of-bias assessment (Figure 3). The prevalent lack of randomization, allocation concealment, and blinding introduces a high risk of selection and performance bias, which likely contributes to the overestimation of treatment effects. This low methodological quality is also a primary driver of the high statistical heterogeneity (*I* ^*2*^ > 70%) observed in our main results. Although our exploratory analyses (Figure 4) suggested that factors such as animal model and timing of administration may contribute to this variability, the limited number of studies precluded a formal meta-regression to quantify these sources. This underscores an urgent need for the field to adopt more rigorous experimental designs and adhere to reporting guidelines, such as ARRIVE, to enhance the reliability and reproducibility of preclinical stroke research.

### 7.4 Highlights and originality

This study is the first to integrate animal experimental evidence of SalA through a systematic review and meta-analysis, clarifying its multi-target effects. Although previous studies have explored the role of *Salvia miltiorrhiza* acid A in ischemic stroke, there is a lack of dedicated systematic reviews or meta-analyses. Therefore, our manuscript fills this gap by systematically summarizing the research progress on *Salvia miltiorrhiza* acid A in this field. Through meta-analysis, we evaluate the effects of different doses and timing of administration on its therapeutic efficacy, providing guidance for clinical application. Additionally, we identify potential biases and shortcomings in the existing studies through quality assessment, offering directions for future research improvements.

### 7.5 Limitations and implications for future research

This meta-analysis has several limitations. First, as discussed, the conclusions are based on primary studies with significant methodological flaws. Second, the lack of systematic safety reporting in the included studies prevented any quantitative assessment of SalA’s adverse effects. Third, although, in our study, Egger’s tests did not detect significant publication bias, this may be due to the limited number of studies, and the potential for bias cannot be entirely ruled out. Consequently, the immediate clinical translation of SalA based on this evidence would be premature. Instead, these findings should serve as a strong impetus for conducting future, higher-quality preclinical studies. Future research should prioritize the following: (1) the use of standardized, blinded, and randomized study designs; (2) perform *a priori* sample size calculations; (3) systematically report on safety and adverse events; and (4) directly compare different dosages and administration windows to define an optimal therapeutic protocol.

## 8 Conclusion

In conclusion, this meta-analysis provides the first quantitative evidence that salvianolic acid A has a significant and multi-faceted neuroprotective effect in animal models of cerebral ischemia. The consistency of this protective signal across various outcomes—despite the considerable heterogeneity and high risk of bias identified in the primary studies—highlights the potential robustness of SalA’s therapeutic action. Crucially, our work not only synthesizes current evidence but also provides an essential roadmap for future research by indicating the specific methodological improvements required to strengthen the evidence base. Therefore, although SalA stands out as a highly promising therapeutic candidate, its successful clinical translation is contingent upon validation through the kind of rigorous, high-quality preclinical studies that this analysis calls for.

## Data Availability

The original contributions presented in the study are included in the article/Supplementary Material; further inquiries can be directed to the corresponding author.

## Glossary

95% CI: 95% confidence interval
AKT: Protein kinase B
ATSM: Autologous Thrombotic Stroke Model
Bcl-2: B-cell lymphoma-2
Bax: Bcl-2-associated X protein
BBB: Blood–brain barrier
Caspase 3: Cysteine–aspartic protease 3
Caspase-9: Cysteine–aspartic protease 9
CIS: Clinical Investigation Study
Cyt-C: Cytochrome C
CCI: Chronic cerebral ischemia
CIRI: Cerebral ischemia/reperfusion injury
DALY: Disability-adjusted life year
EC: Electrocoagulation-induced
H&E: Hematoxylin and eosin
IS: Ischemic stroke
IR: Ischemia/reperfusion
IL-6: Interleukin 6
IL-1β: Interleukin-1β
IV: Intravenous injection
IG: Intragastric gavage
IP: Intraperitoneal injection
JAK: Janus kinase
mRS: Modified Rankin scale
MDA: Malondialdehyde
MCAO: Middle cerebral artery occlusion
NLRP3: NOD-like receptor pyrin domain-containing 3
NIHSS: National Institutes of Health Stroke Scale
NF-κB: Nuclear factor kappa-B
NeuN: Neuronal nuclei antigen
iNOS: Inducible nitric oxide synthase
PI3K: Phosphatidylinositol 3-kinase
p-AKT: Phosphorylated protein kinase B
ROS: Reactive oxygen species
SalA: Salvianolic acid A
SIRT1: Sirtuin 1
SOD: Superoxide dismutase
SMD: Standardized mean difference
SD rat: Sprague–Dawley rat
STAT: Signal transducer and activator of transcription
TUNEL: Terminal urine nucleotide extraction ligation
TNF-α: Tumor necrosis factor-a
TTC: Triphenyl tetrazolium chloride
ZO-1: Zonula occludens-1
