# Supplementary material for: Protective effects of salvianolic acid A on ischemic stroke: A meta-analysis of preclinical studies
Source: Front Pharmacol. 2025 Nov 19;16:1629258. doi: 10.3389/fphar.2025.1629258 (PMC12673838; doi:10.3389/fphar.2025.1629258)
Supplement: Supplementary file 1 [file Supplementaryfile1.docx]

**Supplementary·Table·1.Retrievalstrategies**

| **Data base** | **Number** | **Search termse** |
| --- | --- | --- |
| **Pubmed** | **#1** | ("Salvianolic Acid A"[MeSH Terms]) OR ("SalA"[Title/Abstract]) OR ("Danshensu"[Title/Abstract]) OR ("Salvianolic acid A"[Title/Abstract]) |
|  | **#2** | ("Stroke"[MeSH Terms]) OR ("Brain Ischemia"[MeSH Terms]) OR ("Ischemic Stroke"[Title/Abstract]) OR ("Cerebral Infarction"[Title/Abstract]) OR ("Middle Cerebral Artery Occlusion"[Title/Abstract]) OR ("MCAO"[Title/Abstract]) OR ("Cerebral Ischemia"[Title/Abstract]) |
|  | **#3** | **#1 And #2** |
|  | | |
| **EMBASE** | **#1** | ischemic AND ('stroke'/exp OR 'stroke' OR 'stroke'/exp OR stroke) |
|  | **#2** | acute AND ischemic AND strokes |
|  | **#3** | cryptogenic AND ischemic AND strokes |
|  | **#4** | salvianolic AND acid AND a |
|  | **#5** | dan AND phenolic AND acid AND a |
|  | **#6** | #1 OR #2 OR #3 |
|  | **#7** | #4 OR #5 |
|  | **#8** | **#6 AND #7** |
|  | | |
| **WB of Sci** | **#1** | TS=(ischemic stroke)) OR TS=(Acute Ischemic Strokes)) OR TS=(Cryptogenic Ischemic Strokes) |
|  | **#2** | TS=(Salvianolic acid A)) OR TS=(dan phenolic acid A) |
|  | **#3** | Preprint Citation Index (Exclude – Database) |
|  | **#4** | **#1 And #2 And #3** |
|  | | |
| **Cochrane** | **#1** | MeSH descriptor: [Ischemic Stroke] explode all trees |
|  | **#2** | Cryptogenic Ischemic Strokes |
|  | **#3** | Acute Ischemic Strokes |
|  | **#4** | [mh "Salvianolic Acid A"] OR "SalA":ti,ab,kw OR "Danshensu":ti,ab,kw |
|  | **#5** | #1 OR #2 OR #3 |
|  | **#6** | **#4 AND #5** |

**Database: 5. China National Knowledge Infrastructure (CNKI)**

Field Query

SU (主题) ('丹酚酸A' + 'Salvianolic Acid A' + 'SalA' + '丹参素') AND ('缺血性卒中' + '脑缺血' + '大脑中动脉阻塞' + 'MCAO' + '脑梗死' + '脑梗塞' + 'Ischemic Stroke')

**Database: 6. Wanfang Data**

Field Query

主题 ("丹酚酸A" OR "Salvianolic Acid A" OR "SalA" OR "丹参素") AND ("缺血性卒中" OR "脑缺血" OR "大脑中动脉阻塞" OR "MCAO" OR "脑梗死" OR "脑梗塞" OR "Ischemic Stroke")

**Database: 7. VIP Database**

Field Query

关键词 (丹酚酸A + Salvianolic Acid A + SalA + 丹参素) * (缺血性卒中 + 脑缺血 + 大脑中动脉阻塞 + MCAO + 脑梗死 + 脑梗塞 + Ischemic Stroke)

**Database: 8. China Biomedical Literature Database (CBM)**

Step Query

#1 "丹酚酸A"[不加权:扩展] OR "Salvianolic Acid A"[常用词] OR "SalA"[常用词] OR "丹参素"[常用词]

#2 "缺血性卒中"[不加权:扩展] OR "脑缺血"[不加权:扩展] OR "大脑中动脉阻塞"[常用词] OR "MCAO"[常用词] OR "脑梗死"[常用词] OR "Ischemic Stroke"[常用词]

#3 #1 AND #2

**Supplementary Figure legends**

**Figure 1 Forest plots and subgroup analyses of TNF-α indicators:A Forest plots;B: ubgroup analyses**

**Figure 2 Forest plots and subgroup analyses of IL-6 indicators:A Forest plots;B: ubgroup analyses**

**Figure 3 Forest plots and subgroup analyses of IL-1β indicators:A Forest plots;B: ubgroup analyses**

**Figure 4 Forest plot of the ratio of NeuN and TUNEL expression in cells：A Hippocampus；B Cerebral Cortex;C Ischemic Penumbra**

**Figure 5 Forest plots of Bax/β-actin and Bcl-2/β-actin ratios：A Bax/β-actin;B Bcl-2/β-actin**

**Figure 6 Forest plot of Bcl-2/Bax ratio**

**Figure 7 Forest plots of p-AKT/AKT:A Cerebral Cortex;B Hippocampus**

**Figure 8 Forest plots of Caspase3**

**Figure 9 Forest plots of ZO-1/β-actin**

**Figure 10 Forest plots of Occludin/β-actin**

**Figure 11 Sensitivity analysis: (A) Cerebral infarction area; (B) NDS; (C) Cerebral edema volume**

**
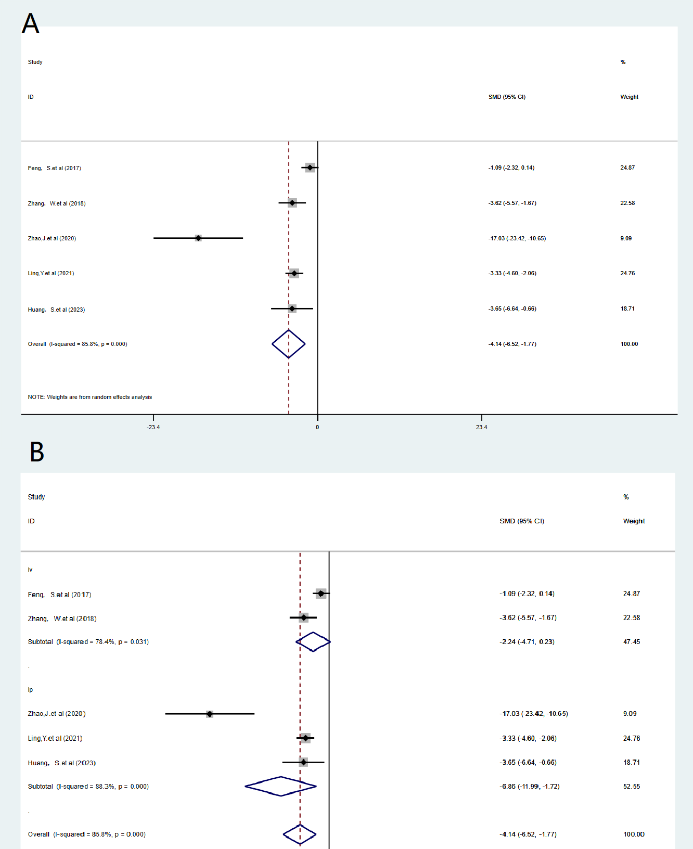
**

**Figure 1 Forest plots and subgroup analyses of TNF-α indicators:A Forest plots**


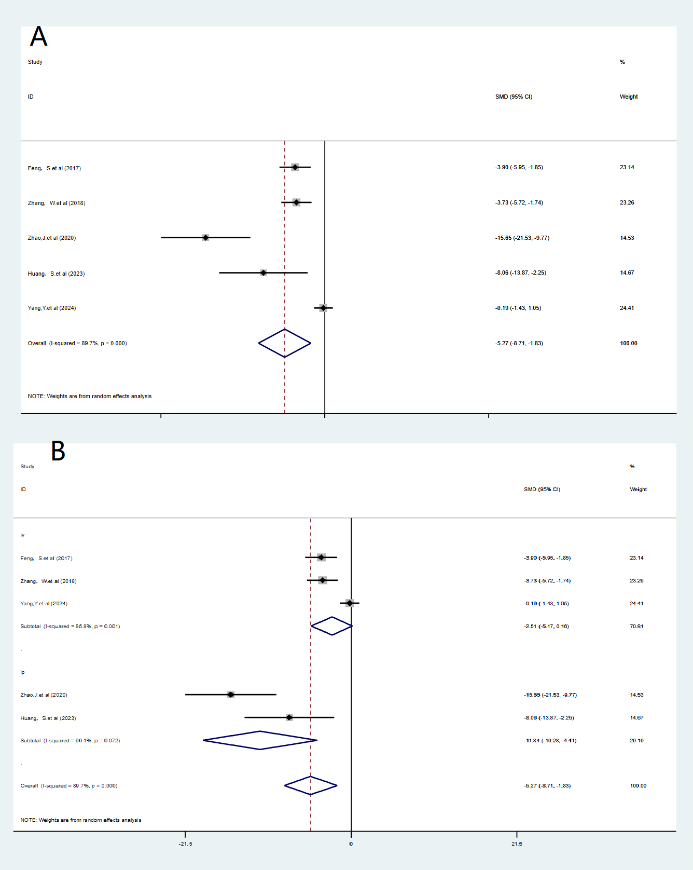


**Figure 2 Forest plots and subgroup analyses of IL-6 indicators:A Forest plots;B: ubgroup analyses**


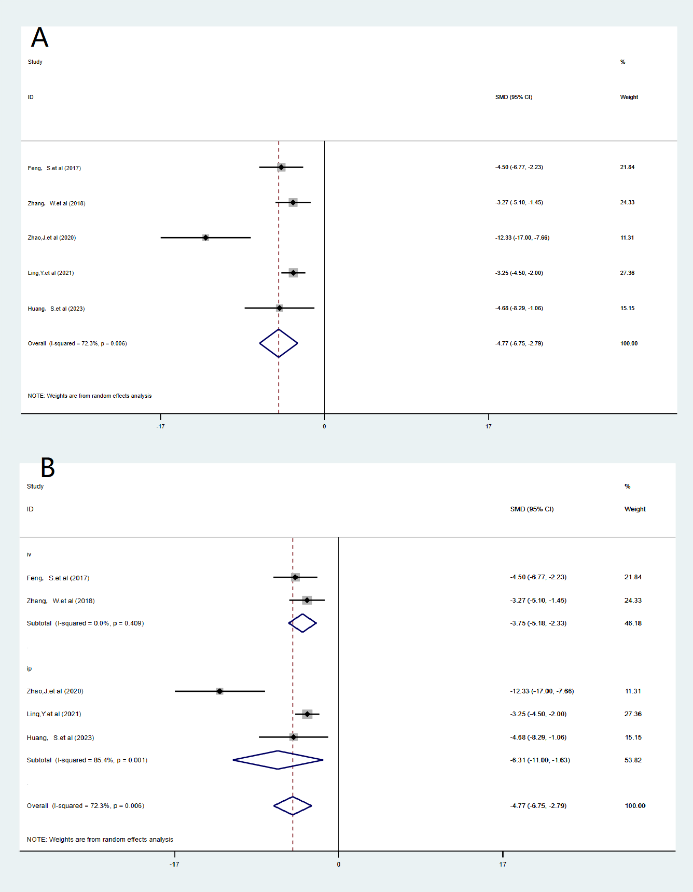


**Figure 3 Forest plots and subgroup analyses of IL-1β indicators:A Forest plots;B: ubgroup analyses**


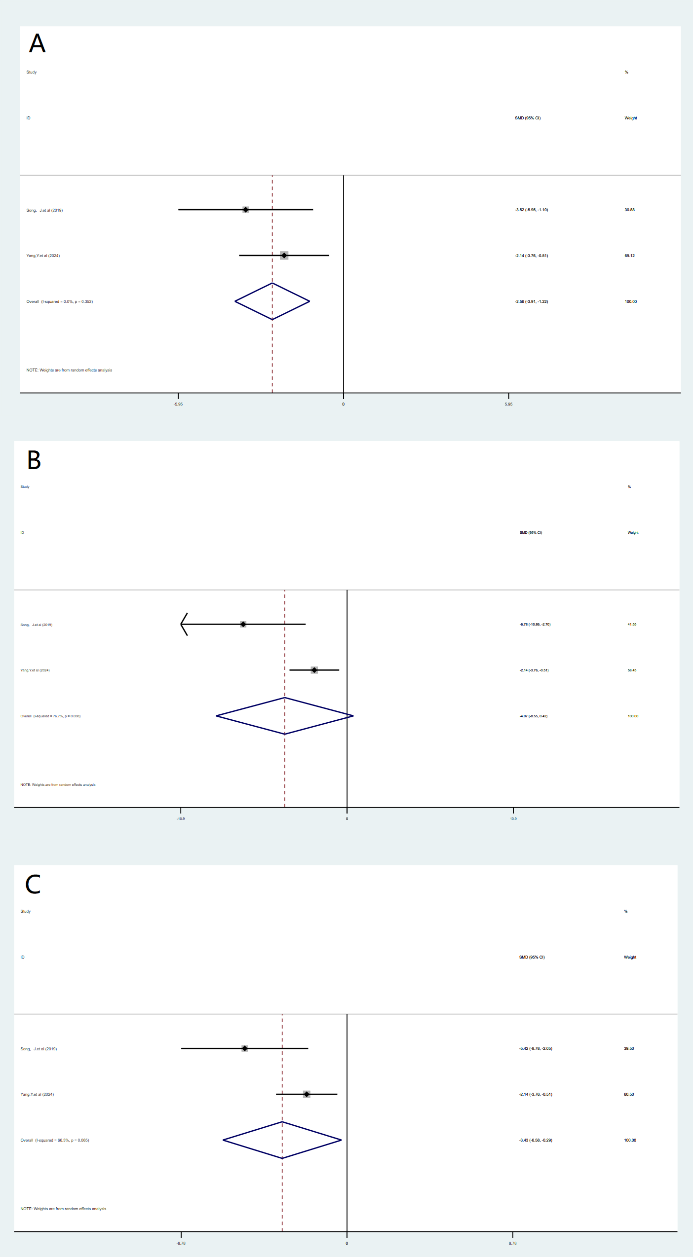


**Figure 4 Forest plot of the ratio of NeuN and TUNEL expression in cells：A** **Hippocampus；B** **Cerebral Cortex;C** **Ischemic Penumbra**


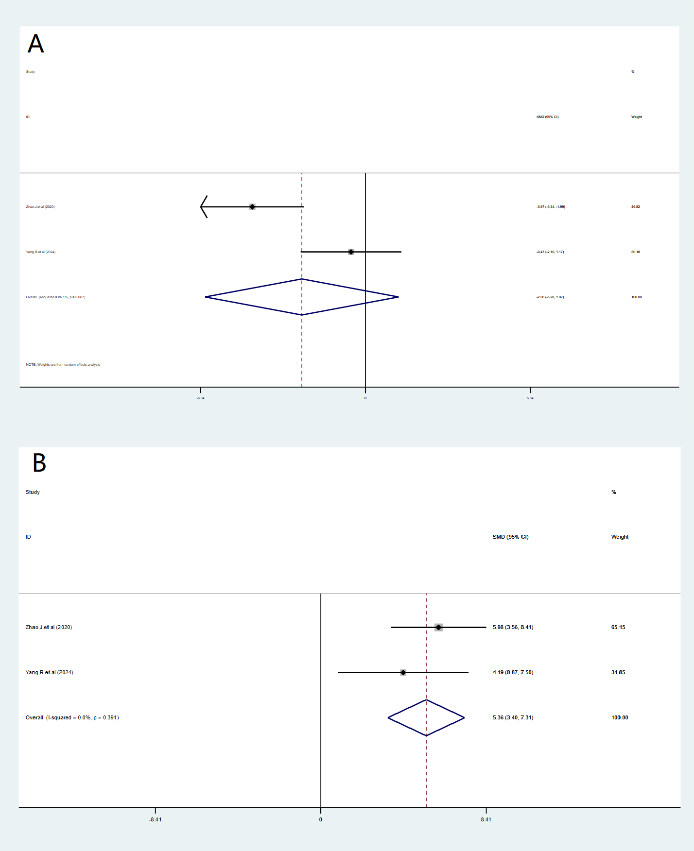


**Figure 5 Forest plots of Bax/β-actin and Bcl-2/β-actin ratios：A Bax/β-actin;B Bcl-2/β-actin**


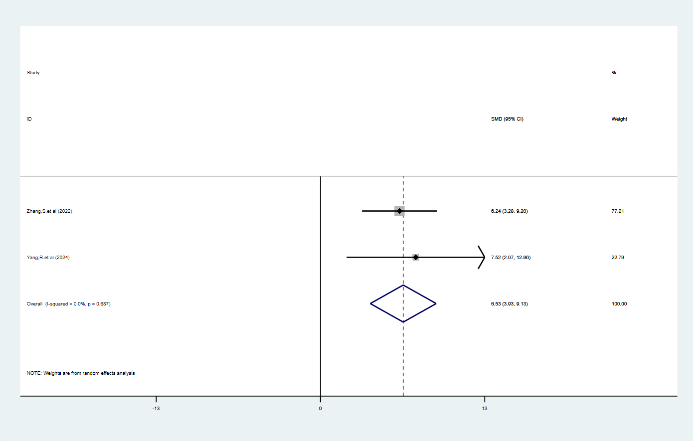


**Figure 6 Forest plot of Bcl-2/Bax ratio**


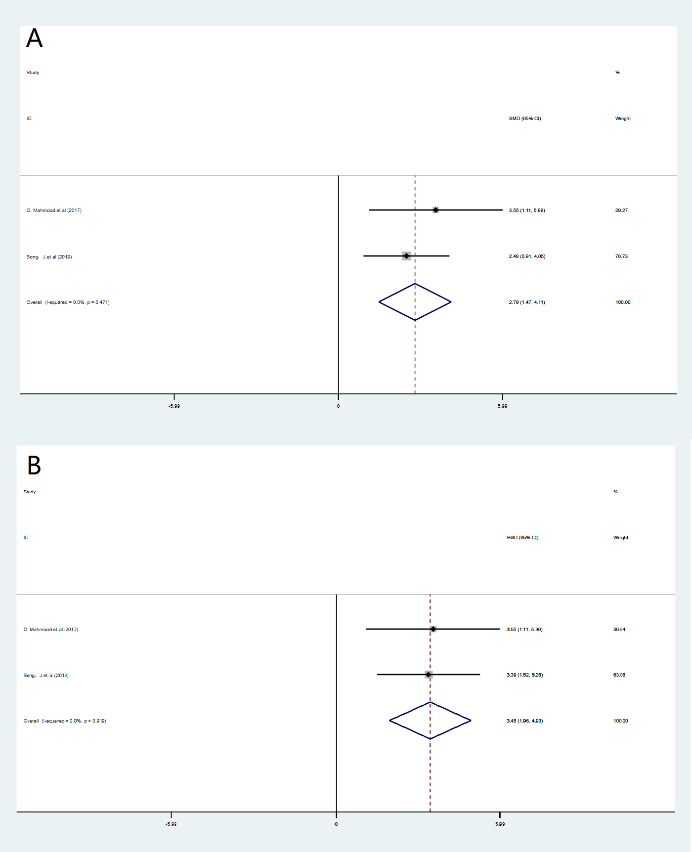


**Figure 7 Forest plots of p-AKT/AKT:A Cerebral Cortex;B Hippocampus**


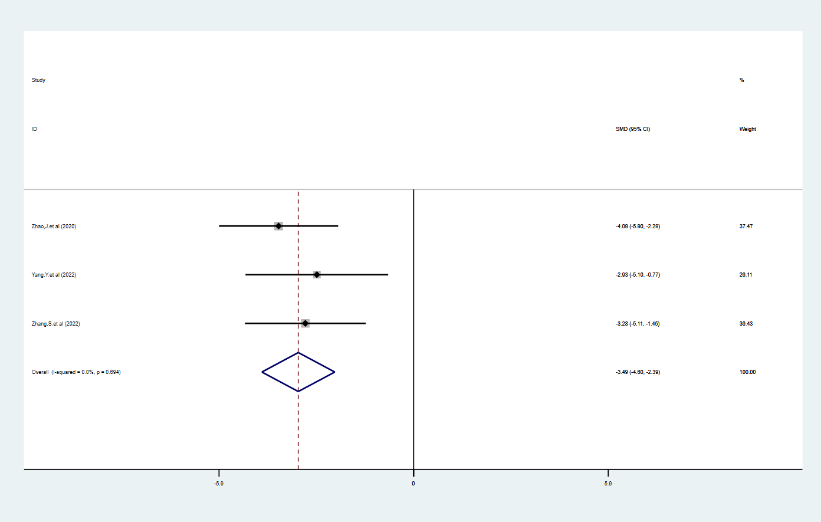


**Figure 8 Forest plots of Caspase3**


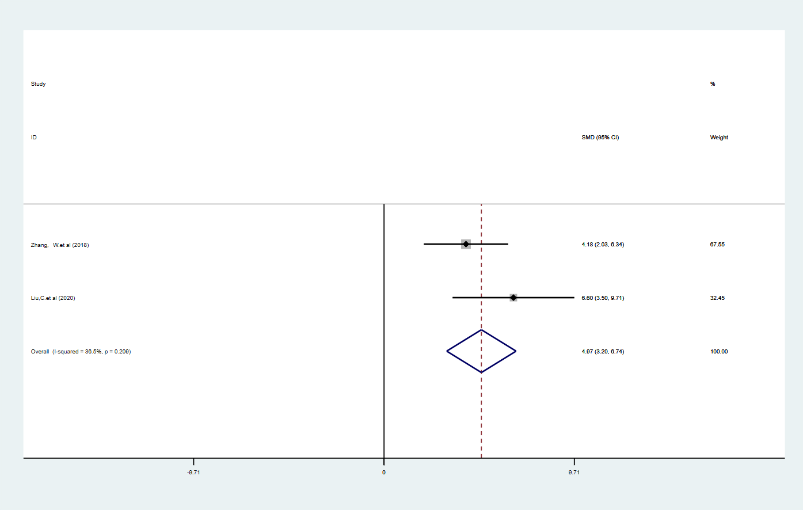


**Figure 9 Forest plots of ZO-1**


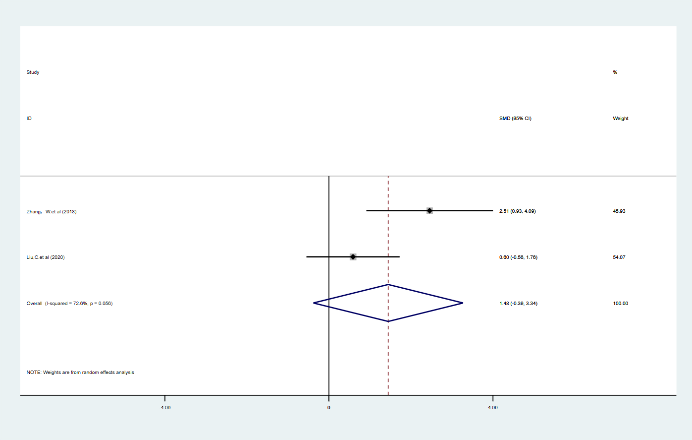


**Figure 10 Forest plots of Occludin/β-actin**


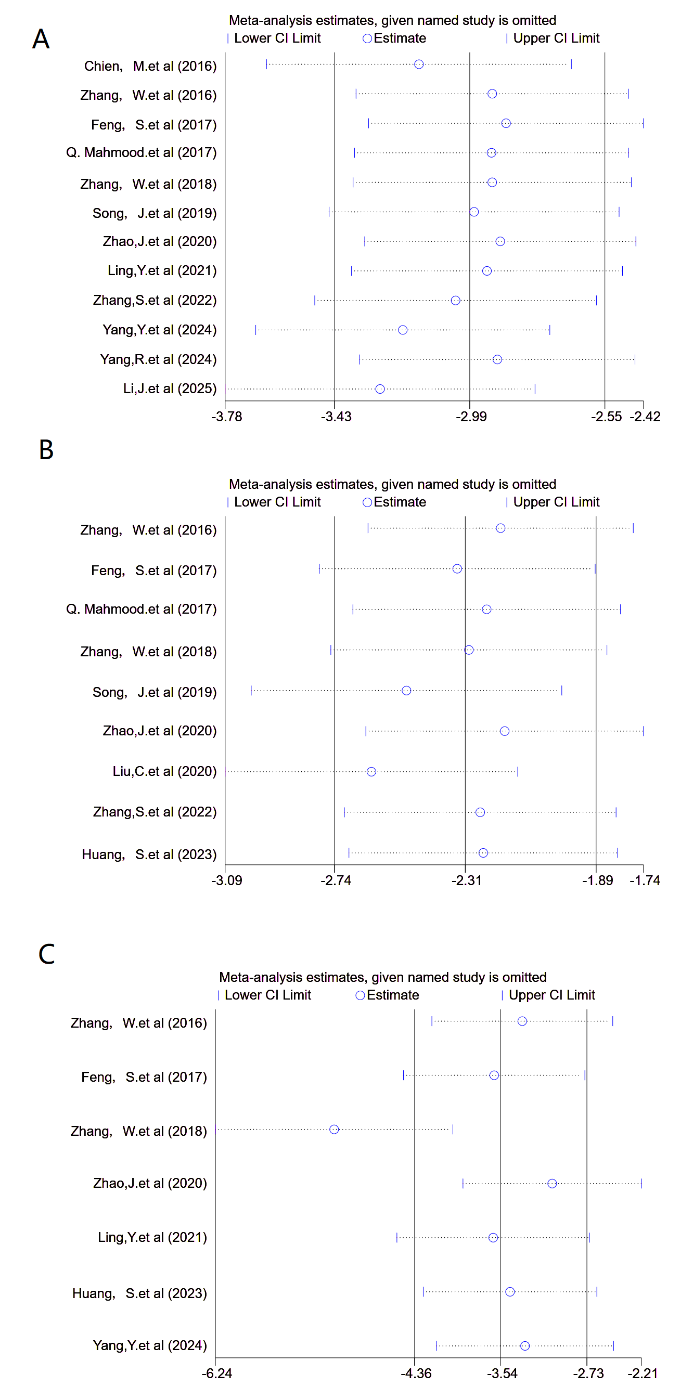


**Figure 11** **Sensitivity analysis: (A) Cerebral infarction area; (B) NDS; (C) Cerebral edema volume**
