# Supplementary material for: Protective effects of salvianolic acid A on ischemic stroke: A meta-analysis of preclinical studies
Source: Front Pharmacol. 2025 Nov 19;16:1629258. doi: 10.3389/fphar.2025.1629258 (PMC12673838; doi:10.3389/fphar.2025.1629258)
Supplement: Supplementary file 2 [file Table1.docx]

| **Section and Topic** | **Item #** | **Checklist item** | **Location where item is reported** |
| --- | --- | --- | --- |
| **TITLE** | | |  |
| Title | 1 | The report is identified as a meta-analysis |  |
| **ABSTRACT** | | |  |
| Abstract | 2 | In the abstract, I have clearly reported the necessary information, including the background, objective, data sources, inclusion criteria, synthesis methods, main results, and conclusions. |  |
| **INTRODUCTION** | | |  |
| Rationale | 3 | In the introduction, it is mentioned that modern medicine has established the standard treatment protocol of intravenous rt-PA thrombolysis combined with endovascular thrombectomy. However, there remains a significant gap in intervention strategies for neuroinflammatory storms and axonal regeneration impairment following reperfusion. Against this background, we have identified that medicinal natural small molecules may offer potential neuroprotective effects in ischemic stroke (IS). Therefore, further investigation into the potential therapeutic role of SalA is of considerable significance. |  |
| Objectives | 4 | My review aims to address the following questions: the molecular mechanisms underlying the neuroprotective effects of SalA in ischemic stroke (IS), and the therapeutic efficacy of SalA in the treatment of IS. |  |
| **METHODS** | | |  |
| Eligibility criteria | 5 | **Inclusion Criteria**: (1) Studies involving rats or mice as subjects, with the establishment of MCAO or I/R models; (2) The experimental group receives SalA intervention post-surgery (selecting the group with the best therapeutic effect if different concentrations are used); (3) The model group receives a placebo or no treatment; (4) No restrictions on animal species, gender, age, weight, or sample size; (5) The primary outcome measures include neurological function scores, infarct area or proportion, and all biomarkers indicating IS.  **The exclusion criteria for the literature are strictly defined as follows:** (1) Non-cerebral ischemia or global cerebral ischemia animal models; (2) Exclusion of reviews, in vitro studies, and trials; (3) Studies where SalA is used to intervene in other diseases or studies that do not use SalA; (4) Studies with insufficient data, including unpublished data; (5) Duplicated studies. The included studies were grouped based on different intervention types, study designs, and outcome measures, and the subsequent data synthesis and analysis were conducted accordingly. |  |
| Information sources | 6 | We searched the following major databases for relevant literature: PubMed, Web of Science, Cochrane Library, Embase, and CNKI (China National Knowledge Infrastructure). The last search was conducted on March 12, 2025. We reviewed reference lists of relevant reviews, systematic reviews, and published studies in the field to ensure no important studies were missed. The last review was conducted in March 2025.All literature searches and data collection were completed in March 2025 to ensure timeliness and accuracy of the information." |  |
| Search strategy | 7 | In the literature search process for this review, we used the following full search strategies:  **PubMed:** (ischemic stroke[MeSH Terms]) OR (ischemic stroke[Title/Abstract])) OR (Acute Ischemic Strokes[Title/Abstract])) OR (Cryptogenic Ischemic Strokes[Title/Abstract])) **And** Salvianolic acid A[MeSH Terms])) OR (Salvianolic acid A[Title/Abstract])) OR (dan phenolic acid A[Title/Abstract])  **EMBASE:** **#1** ischemic AND ('stroke'/exp OR 'stroke' OR 'stroke'/exp OR stroke)**;#2** acute AND ischemic AND strokes **#3** cryptogenic AND ischemic AND strokes**;#4** salvianolic AND acid AND a**;#5** dan AND phenolic AND acid AND a**;#6**#1 OR #2 OR #3**;#7**#4 OR #5**;#8=#6 AND #7**  **WB of Sci:** TS=(ischemic stroke)) OR TS=(Acute Ischemic Strokes)) OR TS=(Cryptogenic Ischemic Strokes) **And** TS=(Salvianolic acid A)) OR TS=(dan phenolic acid A) **And** Preprint Citation Index (Exclude – Database)  **Cochrane:** MeSH descriptor: [Ischemic Stroke] explode all trees OR Cryptogenic Ischemic Strokes OR Acute Ischemic Strokes **And** Salvianolic acid A |  |
| Selection process | 8 | In the selection process, we used the following methods to determine whether a study met the inclusion criteria:  **Screening process**: The screening was conducted by two independent reviewers. Initially, the reviewers screened the titles and abstracts to identify potentially relevant studies. For studies that appeared to meet the inclusion criteria, the full texts were obtained and assessed in detail.  **Independent work**: Both reviewers conducted the screening independently. In cases of disagreement between the reviewers regarding the inclusion of a study, consensus was reached through discussion, or a third-party expert was consulted for the final decision.  **Automation tools**: We used automation tools (EndNote21,) to assist in the initial screening and deduplication of records. These tools helped us efficiently manage the literature and reduce human error, but the final decision on study inclusion was made manually.  **Screening criteria**: During the selection process, we strictly adhered to the inclusion and exclusion criteria mentioned earlier to ensure that all included studies met the review’s requirements." |  |
| Data collection process | 9 | In the data collection process, we employed the following methods:  **1.Data collectors**: Data collection was conducted by two independent reviewers, with each reviewer extracting relevant data from each report. The data extraction process followed a standardized form to ensure consistency and accuracy.  **2.Independent work**: All data collection work was performed independently by both reviewers. In cases of disagreement regarding data extraction or interpretation, the reviewers discussed the discrepancies, and if necessary, a third-party expert was consulted to reach a consensus.  **3.Data verification**: For unclear or uncertain data, we contacted the original study authors to confirm or supplement the data. If the authors were unable to provide additional information, we made reasonable assumptions based on other available data in the study report and documented the relevant assumptions.  **3.Automation tools**: We used automation tools (Excel2020, EndNote21) to help organize and manage the data, ensuring accurate data entry and reducing human error. However, the final data validation and synthesis were manually conducted to ensure the accuracy and consistency of all data." |  |
| Data items | 10a | **Primary outcomes**: Cerebral infarction area, Neurological Deficit Score (NDS), Cerebral edema volume.  **Secondary outcomes**: TNF-α,IL-6, IL-1β, Bax/β-actin, Bcl-2/β-actin, Bcl-2/Bax, Caspase-3, ZO-1/β-actin, Occludin/β-actin, p-Akt/Akt, The ratio of NeuN and TUNEL expression in cells.. |  |
| Study risk of bias assessment | 11 | The risk of bias assessment was conducted using the 10-item CAMARADES checklist proposed by Macleod to evaluate the methodological quality of studies on the effects of Danshensu on IS. The 10 assessment criteria are as follows: (1) sample size calculation, (2) random sequence generation, (3) blinded ischemia induction, (4) blinded outcome assessment, (5) use of anesthetics with no intrinsic neuroprotective effects, (6) appropriate animal models, (7) temperature control declaration, (8) peer-reviewed publication, (9) compliance with animal protection laws, and (10) declaration of potential conflicts of interest. Each study was scored a maximum of 10 points, with 1 point allocated for each criterion. The risk of bias assessment was independently performed by two researchers, and in cases of discrepancies, a third researcher was involved in the assessment to resolve the differences. |  |
| Effect measures | 12 | In my article, for each outcome, I used the Standardized Mean Difference (SMD) to quantify the effects across different results.  **Infarct Volume**: SMD was used to assess the impact of SalA on infarct volume.  **Brain Edema**: SMD was used to evaluate the reduction in brain edema due to SalA treatment.  **Neurological Deficits**: SMD was used to assess the improvement in neurological function after SalA treatment.  **Biomarkers** (e.g., TNF-α, IL-6, Bax/Bcl-2 ratio, Caspase-3): SMD or other appropriate effect measures were used to synthesize the results related to biomarkers. |  |
| Synthesis methods | 13a | In this study, the process of deciding which studies were eligible for each synthesis was carried out through a rigorous screening and evaluation process. First, we conducted an initial screening of all the literature retrieved from four databases (PubMed, Web of Science, Embass，Cochrane Library, and CNKI). Studies that did not meet the predefined inclusion and exclusion criteria were excluded, ensuring that the studies included were relevant to animal models and SalA intervention.  Subsequently, all eligible studies were categorized based on the following intervention characteristics:  **Intervention Methods**: This included the route of administration of SalA (e.g., intravenous injection, intragastric gavage) and dosage (e.g., 20 mg/kg).  **Animal Models**: Studies using models such as middle cerebral artery occlusion (MCAO) or ischemia/reperfusion (I/R).  **Study Outcomes**: These included primary outcome measures like infarct volume, neurological deficit scores, brain edema area, etc.  By comparing these intervention characteristics with the planned groups for each synthesis, we ultimately determined which studies should be included in the meta-analysis, ensuring the scientific rigor and comparability of the results. |  |
|  | 13d | **Meta-analysis Model**: We used the **Standardized Mean Difference (SMD)** as the effect size to measure the impact of SalA on various outcomes across studies. SMD is a commonly used effect size that can handle different measurement scales across studies. For studies with low heterogeneity, we applied a **fixed-effect model**, whereas for studies with high heterogeneity, we used a **random-effects model**. The random-effects model is more suitable for handling the variability between studies in cases of high heterogeneity.  **Statistical Heterogeneity Assessment**: To identify and assess statistical heterogeneity, we used the **I² statistic**. The I² value measures the extent of variability between studies. If the I² value is below 50%, it suggests low heterogeneity and allows for the use of a fixed-effect model. If the I² value is above 50%, it indicates high heterogeneity, and a random-effects model is applied. For all primary outcomes (e.g., infarct volume, brain edema, neurological deficits), we assessed the I² values.  **Software Package**: Data analysis was performed using **STATA 15.0** software. This software supports meta-analysis and provides features to assess heterogeneity, perform sensitivity analysis, and handle missing data, ensuring robust statistical results.  These methods were chosen because they provide reliable effect estimates and help identify and address heterogeneity in the studies, ensuring the scientific rigor and accuracy of the final results. |  |
| Reporting bias assessment | 14 | In this study, to assess the risk of missing results due to reporting biases, we employed the following methods: The use of a **Funnel Plot** to evaluate the presence of systematic bias in small sample studies. An **Egger's test** was conducted on the funnel plot to quantitatively assess the likelihood of reporting bias. Additionally, we performed **sensitivity analysis** by excluding studies that may be affected by reporting bias. |  |
| Certainty assessment | 15 | **GRADE Method**: We applied the **GRADE (Grading of Recommendations, Assessment, Development, and Evaluations)** method to evaluate the certainty of the evidence for each outcome. This method assesses factors such as risk of bias, sample size, study design, consistency of results, and other factors that may affect the reliability of the evidence. In our study, we focused on the following elements:  **①Risk of Bias**: We assessed the quality of the included studies using the CAMARADES checklist, focusing on aspects such as randomization methods, blinding, and sample size calculations to evaluate the risk of bias.  **②Consistency**: We assessed the consistency of results across different studies. For outcomes with significant heterogeneity, we performed subgroup and sensitivity analyses to check if these differences impacted the quality of the evidence.  **③Directness**: We assessed whether the studies directly measured the outcomes of interest rather than indirect inferences.  **④Precision**: We evaluated the precision of the study results, such as the width of confidence intervals, to determine whether the results were statistically significant and reliable.  **Sensitivity Analysis**: We conducted sensitivity analyses to evaluate whether the inclusion or exclusion of certain studies or models changed the strength of the evidence, ensuring the robustness of the conclusions.  By using these methods, we ensured a comprehensive and reliable assessment of the certainty in the evidence for each outcome, making our conclusions more robust and confident. |  |
| **RESULTS** | | |  |
| Study selection | 16a | In the search and selection process for this review, we initially identified **898** records. After removing duplicates and screening out studies that did not meet the inclusion criteria, a total of **15** studies were included in the review. A flow diagram outlining the detailed steps of this process is provided below： 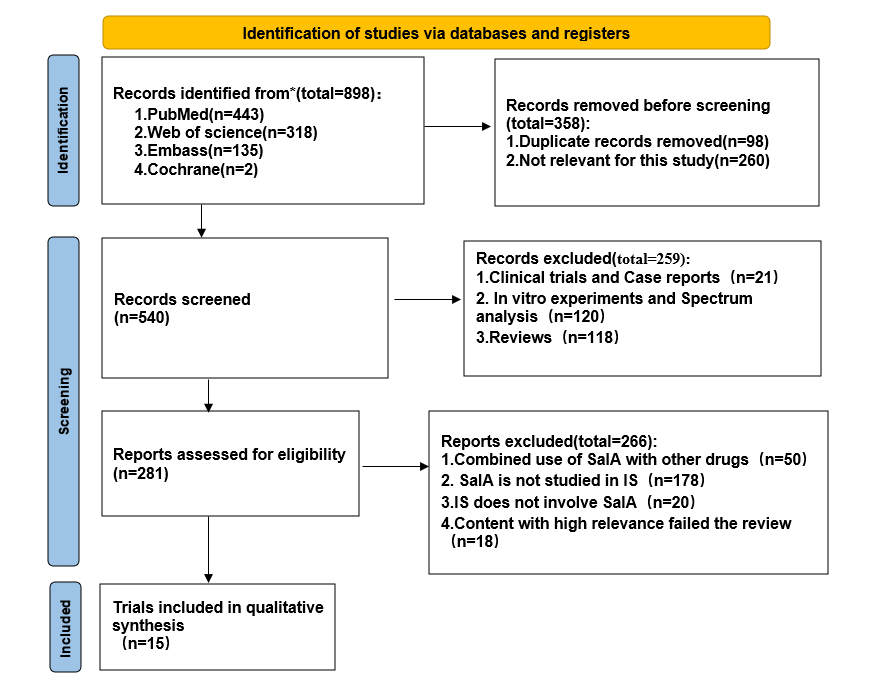 |  |
| Study characteristics | 17 | \| **Study（year）** \| **Species（Sex）** \| **Weight（g）** \| **Animal model** \| **Intervention group(method)** \| **Control group(method)** \| \| --- \| --- \| --- \| --- \| --- \| --- \| \| Chien,M.et al（2016） \| ICR mice（Male） \| 28-30 \| MCAO Model \| 100µg/kg  (Intravenous injection) \| No treatment \| \| Zhang,W.et al（2016） \| SD rats（Male） \| 240-260 \| MCAO/R Model \| 20 mg/kgSalA \| No treatkjkment \| \| Feng,S.et al（2017） \| SD rats（Male） \| 180–200 \| tMCAO Model \| 10 mg/kgSalA  (Intravenous injection) \| No treatment \| \| Q.Mahmood.et al（2017） \| C57BL/6 mice（Male） \| 20-28 \| tMCAO Model \| 5 mg/kg  (Intragastric gavage) \| Treated with saline \| \| Zhang,W.et al（2018） \| SD rats（Male） \| 240-260 \| MCAO Model \| 20 mg/kg  (Intravenous injection) \| Administer equal volume of normal saline \| \| Song,J.et al（2019） \| SD rats（Male） \| 240-260 \| MCAO/R Model \| 20 mg/kg  (Intravenous injection) \| NA \| \| Zhao,J.et al（2020） \| SD rats（Male） \| 250-300 \| MCAO Model \| 20 mg/kg  (Intraperitoneal injection) \| No treatment \| \| Ling,Y.et al（2020） \| SD rats（Male） \| 240-260 \| I/R Model \| 10 mg/kg  (Tail vein injection) \| 1 mg/kg normal saline \| \| Liu,C.et al（2021） \| SD rats（Male） \| 220-260 \| Autologous Thrombotic Stroke Model \| 10 mg/kg  (Intragastric gavage) \| 0.5% CMC-Na, ig \| \| Yang,Y.et al（2022） \| Wistar rat \| 240-260 \| CCI（Two-Vessel Occlusion）Model \| 20 mg/kg \| Treated with saline \| \| Zhang,S.et al（2022） \| SD rats（Male） \| 230-260 \| Electrocoagulation-Induced Autologous Thrombotic Stroke Model \| 10 mg/kg  (Intragastric gavage) \| NA \| \| Huang,S.et al（2023） \| SD rats（Male） \| 250-350 \| MCAO Model \| 20 mg/kg  (Intravenous injection) \| Intraperitoneally injected with 1 mL/kg normal saline \| \| Yang,Y.et al（2024） \| ICR mice/SD rats（Male） \| 16-18/230-240 \| tMCAO Model \| 20 mg/kg  (Intravenous injection) \| same volumes of blank liposomes solution were administered in the same manner \| \| Yang,R.et al（2024） \| SD rats（Male） \| 280-320 \| tMCAO Model \| 8 mg/kg  (Intravenous injection) \| same volume of normal saline \| \| Li,J.et al（2025） \| SD rats（Male） \| 280-320 \| Photothrombotic Model \| 2 mg/kg  (Tail vein injection) \| 100 g/0.1 mL normal saline,iv \| |  |
| Risk of bias in studies | 18 | 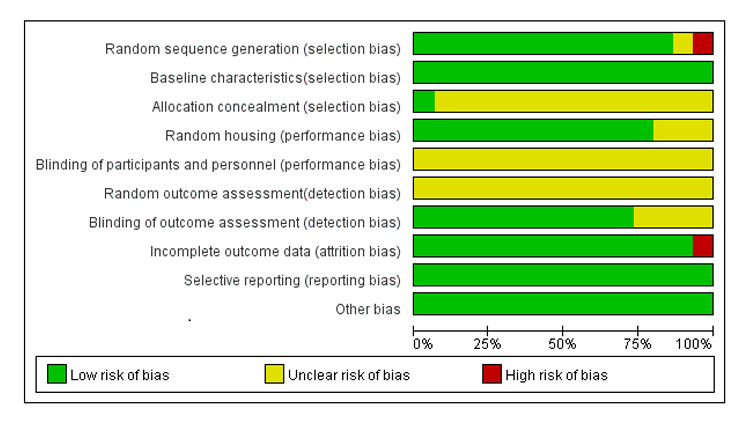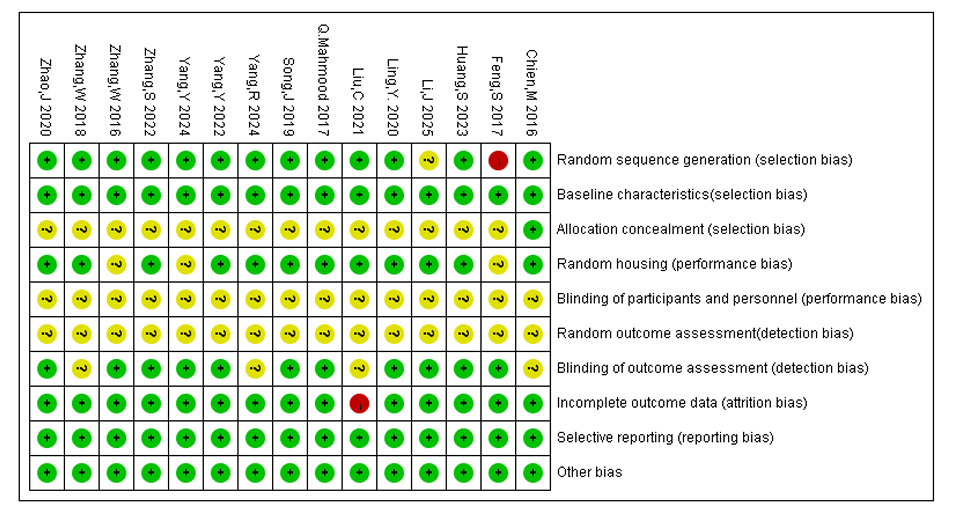 |  |
| Results of individual studies | 19 | Below are a few examples of how the results are presented, typically using structured tables and plots:  **Infarct Volume**:  **Summary Statistics**: For each study, the infarct volume for both experimental and control groups was recorded, including means and standard deviations.  **Effect Estimate**: We used Standardized Mean Difference (SMD) to quantify the effect of SalA on infarct volume. For example, in one study, the experimental group's SMD was -4.67, with a 95% confidence interval of (-5.98, -3.36), p<0.001. 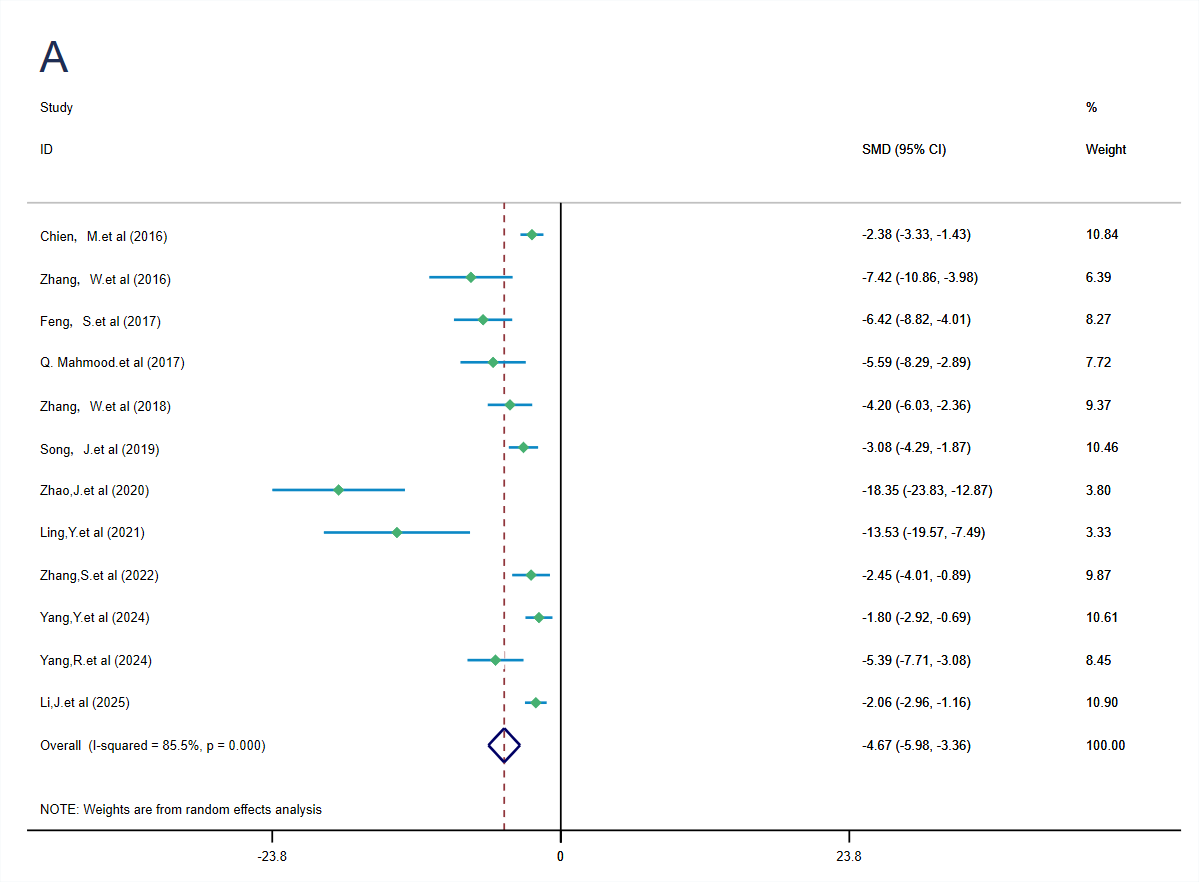 **Brain Edema**:  **Summary Statistics**: Brain edema area was recorded for each group, with means and standard deviations provided.  **Effect Estimate**: The effect size was calculated using SMD. In one study, the experimental group's SMD was -5.291, with a 95% confidence interval of (-7.607, -2.975), p<0.001. 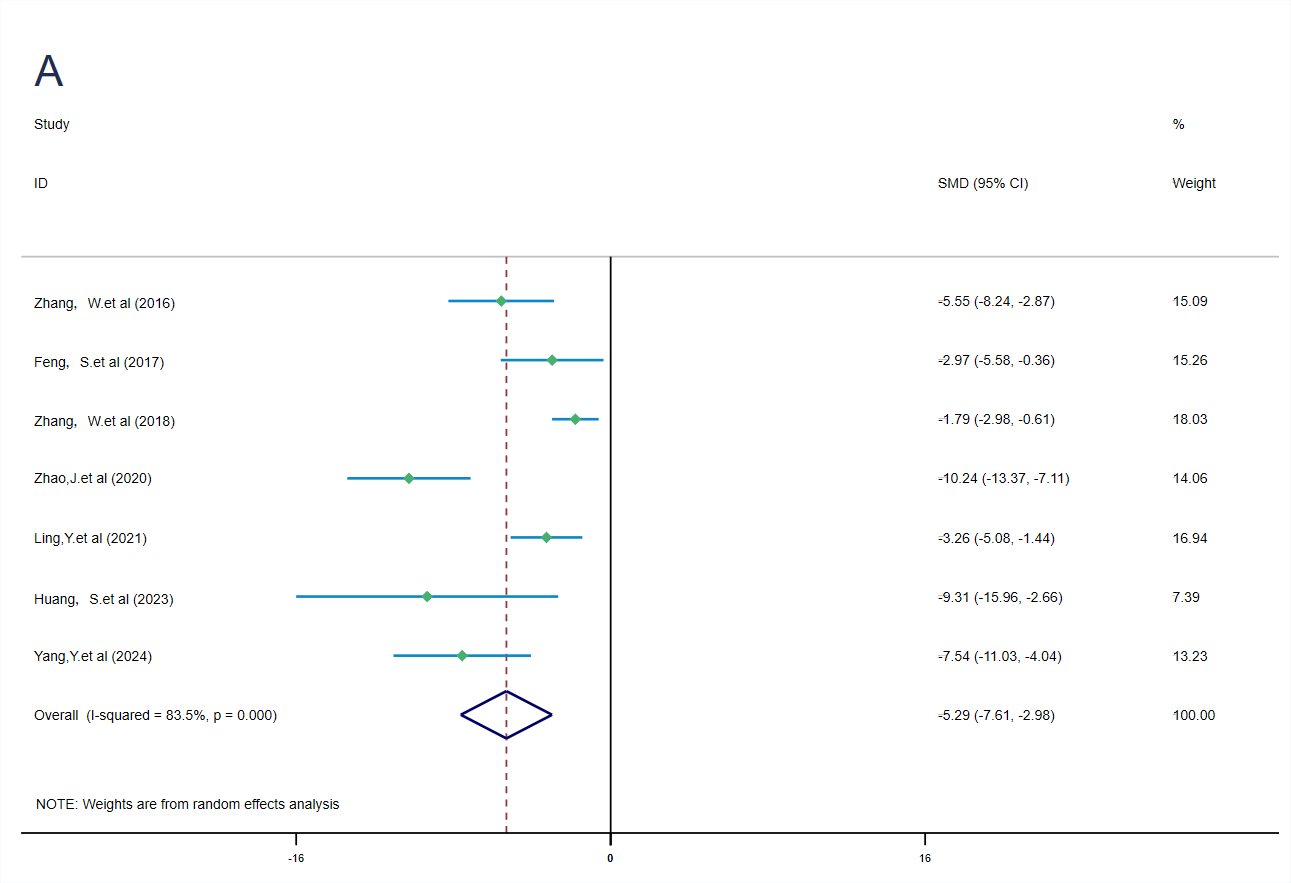 **Neurological Deficits**:  **Summary Statistics**: Each study reported the mean and standard deviation for neurological deficit scores.  **Effect Estimate**: For example, in one study, the SMD for the experimental group was -6.39, with a 95% confidence interval of (-9.091, -3.688), p<0.001. 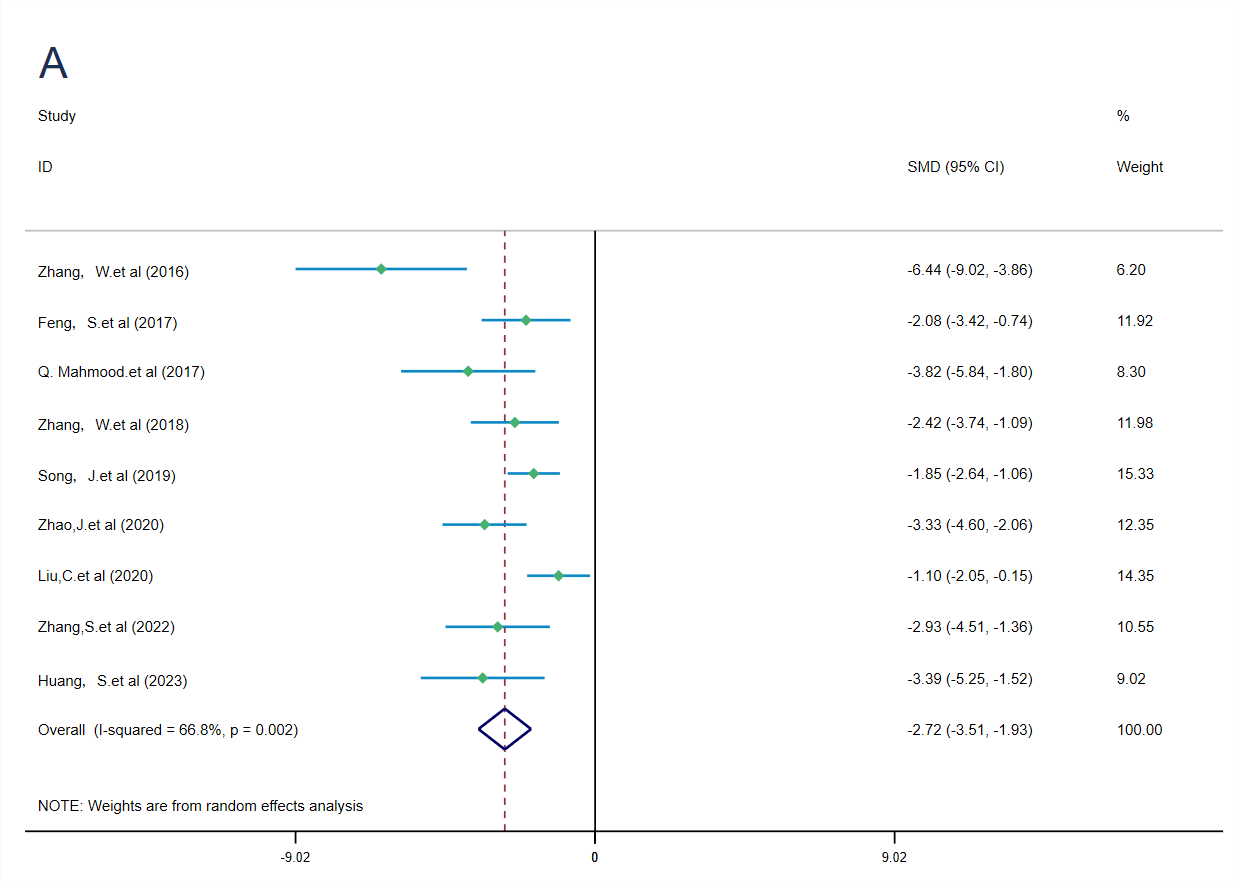 For clarity and comparison, all results are presented in structured tables and visualized using **forest plots**, displaying the effect sizes and their 95% confidence intervals for each study. |  |
| Results of syntheses | 20a | In this study, we summarized the results of each synthesis, including the characteristics of the contributing studies and their risk of bias. Below are summaries for key outcomes:  **1.Synthesis of Infarct Volume**:  **Study Characteristics**: A total of 12 studies contributed to the synthesis of infarct volume, all using animal models (such as MCAO, I/R, etc.) with varying doses and routes of administration of SalA (e.g., intravenous injection, intragastric gavage). The sample sizes ranged from 20 to 130 animals, involving species such as SD rats and ICR mice.  **Risk of Bias**: Most studies met the majority of CAMARADES checklist criteria, including appropriate animal models, experimental design, randomization, and blinding. However, some studies did not explicitly report sample size calculations or blinding assessments, leading to a certain risk of bias. Overall, the risk of bias in these studies was low, making them suitable for meta-analysis. 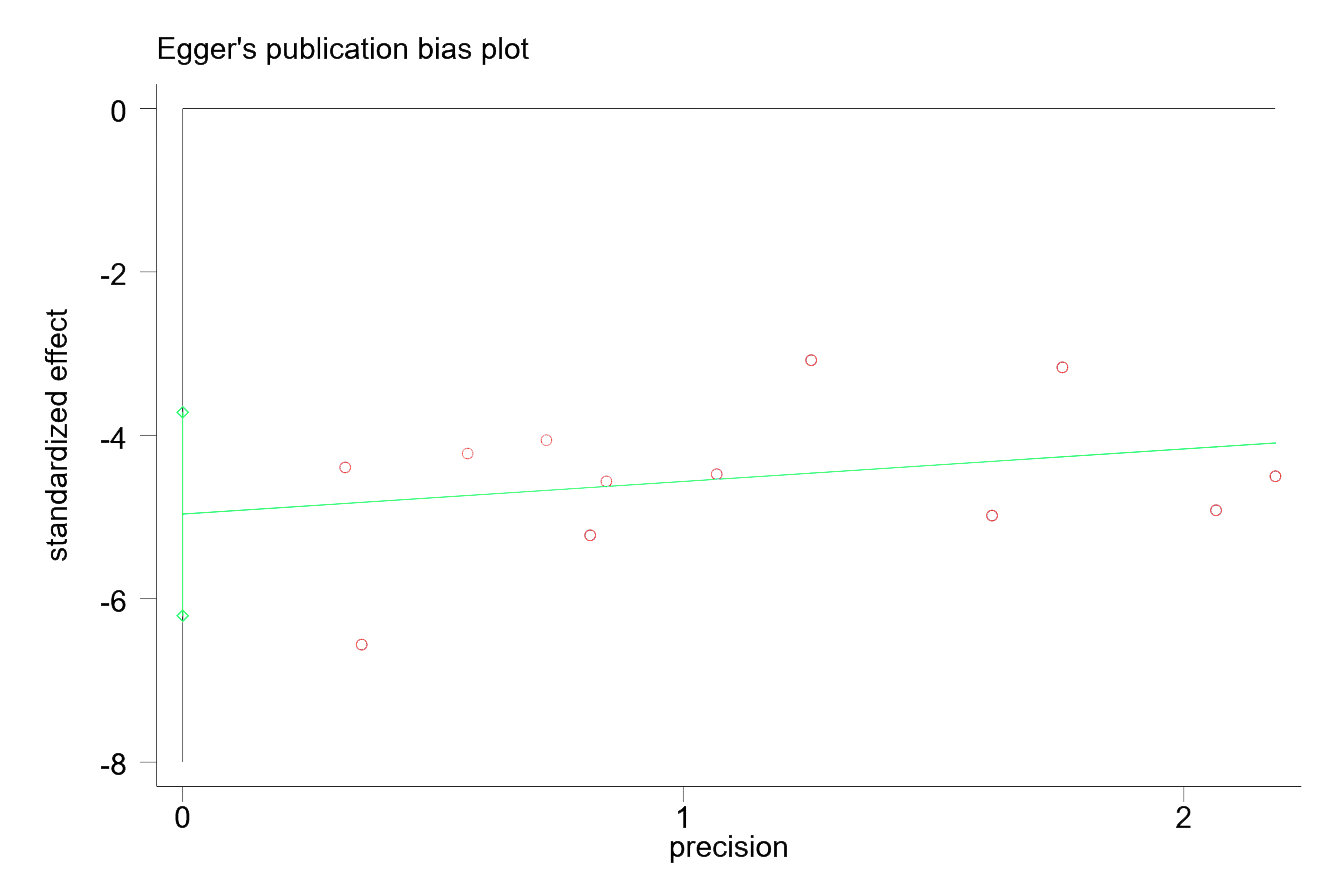 **2.Synthesis of Brain Edema**:  **Study Characteristics**: Seven studies contributed to the synthesis of brain edema, with varying animal models (e.g., MCAO, I/R) and SalA doses and administration routes. The sample sizes ranged from 12 to 60 animals.  **Risk of Bias**: Some studies did not clearly report experimental details (e.g., temperature control, anesthetic selection), which introduced a potential risk of bias. However, most studies adhered to animal ethics and experimental design guidelines, indicating a low risk of bias overall. 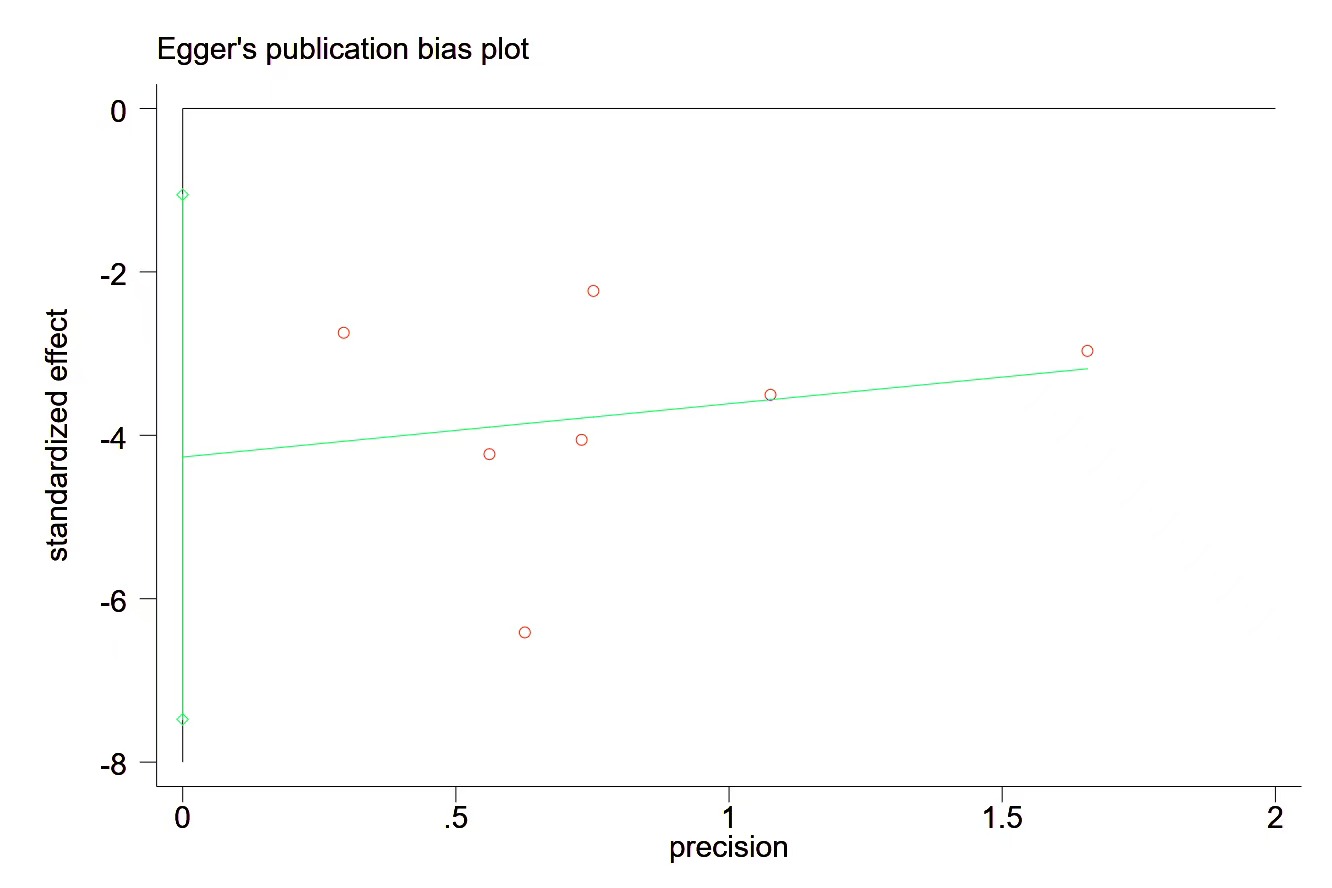 **3.Synthesis of Neurological Deficits**:  **Study Characteristics**: Nine studies contributed to the synthesis of neurological deficits, using different neurological scoring systems and animal models. Sample sizes ranged from 20 to 82 animals, with SalA doses primarily at 10 mg/kg and 20 mg/kg.  **Risk of Bias**: These studies generally used sound experimental designs and control groups, meeting most bias risk assessment standards. A few studies did not fully report blinding or randomization details, which may lead to slight risk of bias. 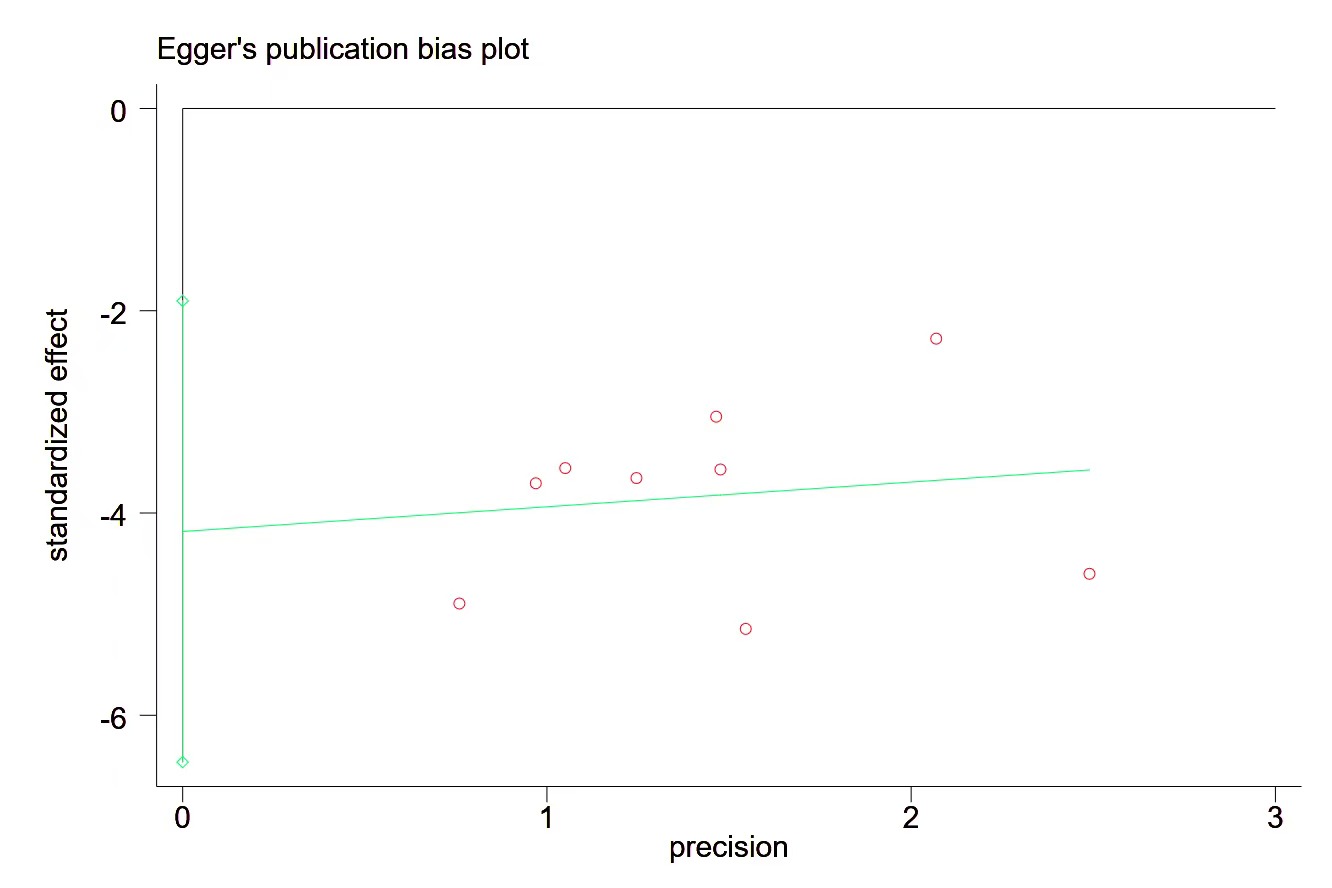 |  |
| Reporting biases | 21 | In this study, we assessed the **risk of bias due to missing results** (reporting biases) for each synthesis. Upon detailed evaluation, we found that none of the outcomes exhibited significant reporting bias. Below are the findings:  **1.Synthesis of Infarct Volume**:  **Reporting Bias Assessment**: The synthesis of infarct volume showed no significant reporting bias.  **Funnel Plot**: The funnel plot was largely symmetrical, with no clear asymmetry, suggesting no significant omission of small or non-significant studies.  **Egger’s Test**: p = 0.08, which is not statistically significant, further confirming that the synthesis was not significantly affected by reporting bias.  **2.Synthesis of Brain Edema**:  **Reporting Bias Assessment**: The synthesis of brain edema also did not show any significant reporting bias.  **Funnel Plot**: The funnel plot was symmetrical, with no noticeable asymmetry, indicating the absence of reporting bias.  **Egger’s Test**: p = 0.056, which is close to significance but not statistically significant, suggesting there was no substantial reporting bias.  **3.Synthesis of Neurological Deficits**:  **Reporting Bias Assessment**: The synthesis of neurological deficits showed no evidence of reporting bias.  **Funnel Plot**: The funnel plot demonstrated symmetrical distribution, with no signs of bias.  **Egger’s Test**: p = 0.112, which is not statistically significant, further confirming the absence of significant reporting bias in this synthesis.  Overall, none of the analyses showed significant reporting bias, indicating a high level of reliability in the synthesized results. |  |
| Certainty of evidence | 22 | **1.Certainty of Evidence for Infarct Volume**:  **Assessment**: We rated the certainty of evidence for infarct volume as **moderate**.  **Reason**: Although all studies used appropriate experimental designs and animal models, and SalA showed significant effects on reducing infarct volume, there was high heterogeneity between studies (I² = 87.8%) and some evidence of publication bias (Egger’s test p = 0.008). These factors slightly impacted the robustness of the evidence, resulting in a moderate rating.  **2.Certainty of Evidence for Brain Edema**:  **Assessment**: For brain edema, we rated the evidence as **moderate**.  **Reason**: Although SalA demonstrated significant effects on reducing brain edema across multiple studies, the presence of moderate heterogeneity (I² = 83.5%) and potential mild publication bias (Egger’s test p = 0.056) impacted the certainty of the evidence, leading to a moderate rating.  **3.Certainty of Evidence for Neurological Deficits**:  **Assessment**: We rated the evidence for neurological deficits as **high**.  **Reason**: In this outcome, all studies adhered to high standards in terms of design and execution, and there was good consistency in effect size estimates (I² = 66.8%). Additionally, funnel plots and Egger’s test did not show significant publication bias (p = 0.112). Thus, we rated the certainty of evidence for neurological deficits as high.  Overall, while some outcomes exhibited certain levels of reporting bias and heterogeneity, the evidence quality for most outcomes was strong, particularly for neurological deficits, which received high confidence. |  |
| **DISCUSSION** | | |  |
| Discussion | 23a | In this study, our results are consistent with findings from other studies. Based on the existing evidence, SalA demonstrates significant benefits in the treatment of IS. However, some studies did not show similar effects, which may be related to factors such as sample size, study design, and intervention methods. Overall, our results further validate [the consensus or discrepancies in existing research], providing important insights for future research. |  |
| **OTHER INFORMATION** | | |  |
| Registration and protocol | 24a | This study has been registered in strict accordance with the PRISMA guidelines and is currently under review for approval. |  |
|  | 24b |  |  |
|  | 24c |  |  |
| Support | 25 | This study was funded by the Key Scientific Research Project of Henan Universities (25A360018), the Science and Technology Tackling Program of Henan Province (222102310529), the Scientific Research Special Project of the National Clinical Research Base of Traditional Chinese Medicine of the Health Commission of Henan Province (2022JDZX005), the Special Project of Cultivation Program for Top Talents in Traditional Chinese Medicine of Henan Province (2022ZYBJ07), the Henan Province Traditional Chinese Medicine Inheritance and Innovation Talent Program (Zhongjing Project) – Leading Talent in Traditional Chinese Medicine (Research-Oriented)(CZ0262-08), and the Mechanistic Study on Acupuncture Combined with Moxibustion Ameliorating Post-Stroke Cognitive Impairment through Regulation of the TLR4 Signaling Pathway (20-21ZY1009). |  |
| Competing interests | 26 | The authors declare that the research was conducted in the absenceof any financial or commercial relationships that could be construed as apotential conflict of interest |  |
| Availability of data, code and other materials | 27 | We adhered to rigorous data management and transparency principles to ensure the accessibility of relevant materials. In addition, supplementary materials have been prepared, and we are able to provide the raw data collected upon request. |  |

*From:*  Page MJ, McKenzie JE, Bossuyt PM, Boutron I, Hoffmann TC, Mulrow CD, et al. The PRISMA 2020 statement: an updated guideline for reporting systematic reviews. BMJ 2021;372:n71. doi: 10.1136/bmj.n71. This work is licensed under CC BY 4.0. To view a copy of this license, visit <https://creativecommons.org/licenses/by/4.0/>
